# Protein signatures of centenarians and their offspring suggest centenarians age slower than other humans

**DOI:** 10.1111/acel.13290

**Published:** 2021-01-29

**Authors:** Paola Sebastiani, Anthony Federico, Melody Morris, Anastasia Gurinovich, Toshiko Tanaka, Kevin B. Chandler, Stacy L. Andersen, Gerald Denis, Catherine E. Costello, Luigi Ferrucci, Lori Jennings, David J. Glass, Stefano Monti, Thomas T. Perls

**Affiliations:** ^1^ Institute for Clinical Research and Health Policy Studies Tufts Medical Center Boston MA USA; ^2^ Bioinformatics Program Boston University Boston MA USA; ^3^ Division of Computational Biomedicine Department of Medicine Boston University School of Medicine Boston MA USA; ^4^ Novartis Institutes for Biomedical Research Cambridge MA USA; ^5^ Translational Gerontology Branch National Institute on Aging Baltimore MD USA; ^6^ Translational Glycobiology Institute Department of Translational MedicineFlorida International University Herbert Wertheim College of Medicine Miami FL USA; ^7^ Geriatric Section Department of Medicine Boston University School of Medicine and Boston Medical Center Boston MA USA; ^8^ Department of Medicine BU‐BMC Cancer Center Boston University School of Medicine Boston MA USA; ^9^ Department of Biochemistry Center for Biomedical Mass Spectrometry Boston University School of Medicine Boston MA USA; ^10^ Regeneron Pharmaceuticals Tarrytown NY USA

**Keywords:** aging, longevity, protein, senescence, SomaLogic

## Abstract

Using samples from the New England Centenarian Study (NECS), we sought to characterize the serum proteome of 77 centenarians, 82 centenarians' offspring, and 65 age‐matched controls of the offspring (mean ages: 105, 80, and 79 years). We identified 1312 proteins that significantly differ between centenarians and their offspring and controls (FDR < 1%), and two different protein signatures that predict longer survival in centenarians and in younger people. By comparing the centenarian signature with 2 independent proteomic studies of aging, we replicated the association of 484 proteins of aging and we identified two serum protein signatures that are specific of extreme old age. The data suggest that centenarians acquire similar aging signatures as seen in younger cohorts that have short survival periods, suggesting that they do not escape normal aging markers, but rather acquire them much later than usual. For example, centenarian signatures are significantly enriched for senescence‐associated secretory phenotypes, consistent with those seen with younger aged individuals, and from this finding, we provide a new list of serum proteins that can be used to measure cellular senescence. Protein co‐expression network analysis suggests that a small number of biological drivers may regulate aging and extreme longevity, and that changes in gene regulation may be important to reach extreme old age. This centenarian study thus provides additional signatures that can be used to measure aging and provides specific circulating biomarkers of healthy aging and longevity, suggesting potential mechanisms that could help prolong health and support longevity.

## Graphical abstract

Comparison of the serum proteome of centenarians, their offspring and controls without familial longevity shows that centenarian serum is enriched for some proteins of aging and for distinct protein signatures of extreme longevity. In particular, our analyses suggest that centenarians acquire similar aging signatures as seen in younger cohorts that have shorter survival periods, suggesting that centenarians do not escape normal aging markers, but rather acquire them much later than usual.

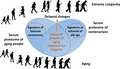

## 1 INTRODUCTION

The increased life expectancy of humans is an important public health accomplishment that has contributed to the increased prevalence of older individuals in the population. Older age is considered the most important risk factor for Alzheimer's disease, cancer, cardiovascular disease, dementia, influenza, and neuro‐degenerative disorders. However, an important lesson learned from studying centenarians and their offspring is that there are factors that protect some individuals from conditions associated with older ages (Andersen et al., 2012; Ismail et al., 2016; Partridge et al., 2018; Sebastiani et al., 2013). Given the finding that centenarians seem relatively protected from age‐related conditions, it is naturally of interest to study genetic and molecular profiles of both centenarians and their offspring to determine the genetic basis as to how and why some people age more healthily than others, in an effort to ultimately discover treatments for age‐related disorders (Kaeberlein et al., 2015; Schork et al., 2018; Sebastiani & Perls, 2012).

Genetic association studies are identifying genes and genetic signatures that predict extended lifespan and health span, although the biological mechanisms that link genotypes to phenotypes remain elusive even for most replicated associations (Broer et al., 2015; Sebastiani, Gurinovich, et al., 2017; Sebastiani et al., 2012; Timmers et al., 2019). Studies in humans and other species are identifying molecular signatures of aging and lifespan that use tissue‐specific transcriptional profiles, DNA methylation, metabolomics, and protein profiles to characterize molecular mechanisms linked to aging and related diseases that could mediate the genotype to phenotype associations (Eline Slagboom et al., 2018; Horvath, 2013; Orwoll et al., 2018; Peters et al., 2015; Sebastiani, Thyagarajan, et al., 2017; Shavlakadze et al., 2019; Singh et al., 2019; Tanaka et al., 2018; Tian et al., 2017; Timmons, 2017).

In recent years, serum and plasma proteins have emerged as powerful circulating biomarkers of aging and disease from easily accessible biological samples, and the SomaLogic technology has provided a reliable tool to measure a large number of circulating proteins with high reproducibility (Candia et al., 2017; Davies et al., 2012; Hathout et al., 2015; Tanaka et al., 2018). Recent studies have shown that both plasma and serum proteins, when measured on a large scale, overlap with tissue‐specific transcriptional profiles and can be used to link genotypes to phenotypes to suggest druggable targets (Sun et al., 2018). Many circulating proteins also appear to overlap with tissue‐specific markers of cell senescence (Basisty et al., 2019). In addition, the analysis of protein profiles in serum has revealed clusters of co‐regulated proteins that can be used to annotate the biological mechanisms underlying disease and aging (Emilsson et al., 2018). All these results suggest that serum and plasma provide easy access to protein data that can be used not only as diagnostic and prognostic biomarkers but also to implicate biological mechanisms.

Here, we describe a comprehensive proteome scan of serum from centenarians, centenarians' offspring, and unrelated controls, age‐matched to the offspring but without familial longevity, that we generated to characterize the serum proteome of centenarians and to discover proteins and their change patterns that may be important for longevity and healthy aging. We first show that there is a large number of proteins in the serum of centenarians whose expression changes compared with younger individuals. By comparing this set with those published in other studies of aging, we show that centenarian serum is enriched of highly expressed aging proteins, including, for example, biomarkers of cell senescence, but also of proteins that may be either the cause or the consequence of extreme longevity. These cross‐sectional data, however, do not capture the changes that occurred in the serum profiles of centenarians at a much younger age and that may have determined their extreme longevity. Therefore, we use proteins that correlate with survival to show that delayed changes of many proteins of aging correlate with longer survival and that key protein biomarkers of longer survival change with older age. To advance a system‐level investigation of potential mechanisms of healthy aging, we also move beyond one‐protein‐at‐a‐time analyses—where differential analyses are performed to rank proteins or pathways based on their differential behavior between the groups—and we apply a co‐expression network analysis approach to capture global expression patterns and the molecular rewiring that differ between centenarians and offspring and controls.

## 2 RESULTS

Table 1 summarizes the demographic characteristics of the 73 healthy centenarians and 4 nonagenarians (age at blood draw 92–114 years), 82 centenarians' offspring (age at blood draw 45–91 years), and 65 controls (age at blood draw 52–88 years) that we selected for this study. For simplicity, we will refer to the first group of 77 participants as centenarians. Participants were enrolled between 2003 and 2016 in the New England Centenarian Study (NECS), using the same protocol for blood collection and serum storage. The protein profiles of the serum samples were generated using a Novartis custom‐designed SomaScan platform that profiles 4785 aptamers corresponding to 4116 unique proteins. Based on quality control analyses described in the methods section and Figure S1, we removed two outlier samples and analyzed the data of the remaining 224 samples in a variety of ways to identify protein signatures of aging, extreme old age, and survival. We conducted a series of analyses that are summarized in the flowchart in Figure S2 to (a) discover serum proteins whose expression changes between centenarians, offspring, and controls, and investigate their relation to protein biomarkers of aging; (b) discover proteins that correlate with survival, and how they relate to protein biomarkers of aging and to the proteins expressed in centenarians; and (c) discover clusters of co‐expressed proteins that can point to different processes in the serum of centenarians compared with the younger individuals.

**TABLE 1:** Patients' characteristics

	Centenarians	Offspring	Controls
Number	77	82	65
Mean age at serum years (*SD*)	105.7 (3.6)	71.2 (9.3)	70.6 (7.8)
Mean age at last contact (*SD*)	107.9 (3.5)	83.5 (8.8)	81.5 (7.0)
% alive (as of 01/2019)	6%	77%	82%
% Female	66%	66%	55%

### 2.1 Protein signature of centenarians

#### 2.1.1 Single protein analysis

**FIGURE 1:**
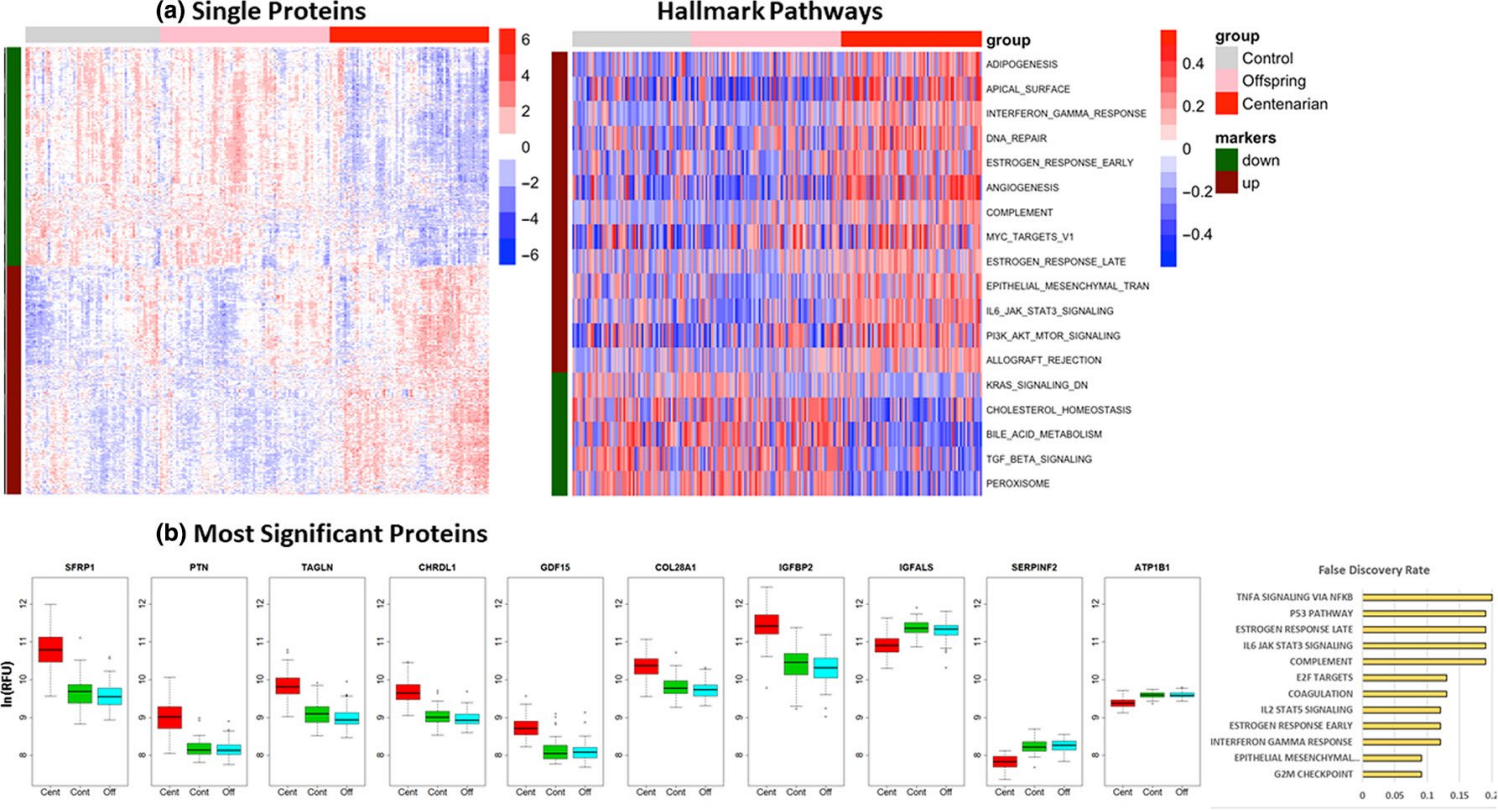
Proteomic signatures of centenarians. (a) *Left*: Heatmap of the 1428 SomaScan aptamers (1312 proteins) that were significantly different between centenarians, centenarians' offspring, and controls at 1% false discovery rate (FDR). The rows represent aptamers grouped into those lower in centenarians compared with the other two groups (695 aptamers, green bar on the left), and those higher in centenarians compared with the other groups (733 aptamers, red bar on the left). The columns represent samples, grouped into controls (gray bar on the top), centenarians' offspring (pink bar on the top), and centenarians (red bar on the top). The values in each cell represent log‐transformed relative fluorescence unit (RFU), standardized by row. The rows were clustered within up‐ and downregulated groups, and the columns were clustered within centenarians, centenarians' offspring, and controls using hierarchical clustering. *Right*: Heatmap of the 18 Hallmark pathways that were significantly altered in centenarians, centenarians' offspring, and controls at 5% FDR. The rows represent pathways, grouped into those downregulated in centenarians (5 pathways, green band on the left), and those upregulated in centenarians compared with the other two groups (13 pathways, red band on the left). The values in each cell represent the pathway scores, standardized by row. (b) Selection of 10 proteins with differential intensity in centenarians (red boxplots), controls (green), and centenarian offspring (cyan). Values on the *y*‐axis are log‐transformed relative fluorescence unit (RFU). The barplot in orange shows the adjusted *p*‐values of the 12 pathways enriched in the set of 1312 differentially expressed proteins

We correlated the protein levels to the groups corresponding to centenarians, centenarians' offspring, and controls using an ANOVA type analysis, adjusted by sex and year of sample collection. The analysis identified 1428 aptamers corresponding to 1312 unique proteins with expression that significantly differed in centenarians with a 1% false discovery rate (FDR). No aptamer reached a statistically significant difference in the comparison of centenarians' offspring and controls, after adjusting for multiple comparisons, thus indicating that the major distinguishing feature was the extreme old age of centenarians. The heatmap in Figure 1a shows clear patterns of expression of the 1428 aptamers that distinguish the serum protein profiles of centenarians from the serum protein profiles of centenarians' offspring and controls. The protein targets of 733 aptamers were over‐expressed in centenarians' serum (median fold change and range comparing centenarians to controls: 1.26 (1.02; 3.33); median fold change and range comparing centenarians to offspring: 1.25 (1.02; 3.33)), and the protein targets of 695 aptamers were under‐expressed in centenarians' serum (median fold change and range comparing centenarians to controls and to offspring: 0.88 (0.56; 0.97)). Table 2 describes the top 30 protein targets detected as differentially expressed, while the full set of results is in Table S1a. Figure 1b displays the patterns of protein expression levels (measured in log(RFU)) of some of the most significant results. The set includes known proteins of aging such as the growth differentiation factor 15 (GDF15) that was the protein most significantly correlated with age in a recently published plasma protein signature of aging (Tanaka et al., 2018), pleiotrophin (PTN), and chordin‐like 1 (CHRDL1), previously associated with aging with consistent effects (Menni et al., 2015; Tanaka et al., 2018), and insulin growth factor‐binding protein 2 (IGFBP2) (Tanaka et al., 2018). The centenarian signature also included 32 of the 75 candidate biomarkers of aging that were examined by the TAME biomarker working group (Justice et al., 2018) and were represented in the SomaScan platform. This set includes the growth differentiation factor 11 (GDF11) that was under‐expressed in centenarians consistently with the previously reported decline with age (Egerman & Glass, 2019), C‐reactive protein, cystatin C, growth hormone receptor, insulin‐like growth factor receptor, and many more. The full list is included in Table S1b. Figure 1b also includes examples of proteins such as secreted frizzled‐related protein 1 (SFRP1), a potential biomarker of the aging of cardiac stem/progenitor cells (Nakamura & Hoppler, 2017), transgelin (TAGLN), and collagen type XXVIII alpha 1 chain (COL28A1) that have not been previously linked to aging and are over‐expressed in centenarians' serum. The figure also includes three examples of proteins that have lower levels in centenarians' serum compared with younger individuals: insulin‐like growth factor‐binding protein acid‐labile subunit (IGFALS), serpin family F member 2 (SERPINF2), and the glycoprotein ATPase Na+/K+ transporting subunit beta 1 (ATP1B1). Sixty‐eight of these proteins (Table S1c) overlapped with genes whose expression correlates with age in the transcriptional profile of human peripheral blood (Peters et al., 2015).

**TABLE 2:** The top 30 differentially expressed proteins between centenarians and centenarians' offspring and controls

SomaScan ID	UniProt	Gene symbol	Gene name	FC cont to cent	FC off to cent	P F test	Brief description
14042‐11_3	Q8N474	SFRP1	Secreted frizzled‐related protein 1	0.30	0.27	7.91E‐35	This gene encodes a member of the SFRP family that contains a cysteine‐rich domain homologous to the putative Wnt‐binding site of frizzled proteins. Members of this family act as soluble modulators of Wnt signaling
3045‐72_2	P21246	PTN^a^	Pleiotrophin	0.42	0.41	3.88E‐34	The protein encoded by this gene is a secreted heparin‐binding growth factor. The protein has significant roles in cell growth and survival, cell migration, angiogenesis, and tumorigenesis
9756‐6_3	Q01995	TAGLN	Transgelin	0.46	0.41	2.74E‐30	The protein encoded by this gene is a transformation and shape‐change sensitive actin cross‐linking/gelling protein found in fibroblasts and smooth muscle
3362‐61_2	Q9BU40	CHRDL1^a^	Chordin‐like 1	0.53	0.50	1.43E‐29	An antagonist of bone morphogenetic protein 4. The encoded protein may play a role in topographic retinotectal projection and in the regulation of retinal angiogenesis in response to hypoxia
8304‐50_3	O00300	TNFRSF11B	TNF receptor superfamily member 11b	0.53	0.53	6.50E‐27	The protein encoded by this gene is a member of the TNF receptor superfamily. This protein is an osteoblast‐secreted decoy receptor that functions as a negative regulator of bone resorption
3234‐23_2	Q76M96	CCDC80^a^	Coiled‐coil domain containing 80	0.60	0.56	2.87E‐26	Predicted cellular protein (secreted)
4374‐45_2	Q99988	GDF15^a^	Growth differentiation factor 15	0.52	0.51	5.28E‐25	This gene encodes a secreted ligand of the TGF‐beta superfamily of proteins. Ligands of this family bind various TGF‐beta receptors leading to recruitment and activation of SMAD family transcription factors that regulate gene expression. Increased protein levels are associated with disease states such as tissue hypoxia, inflammation, acute injury, and oxidative stress
8819‐3_3	P18065	IGFBP2	Insulin‐like growth factor‐binding protein 2	0.37	0.35	7.53E‐25	The protein encoded by this gene is one of six similar proteins that bind insulin‐like growth factors I and II (IGF‐I and IGF‐II). The encoded protein can be secreted into the bloodstream, or it can remain intracellular. High expression levels of this protein promote the growth of several types of tumors and may be predictive of the chances of recovery of the patient
11109‐56_3	Q4LDE5	SVEP1^+^	Sushi, von Willebrand factor type A, EGF and pentraxin domain	0.56	0.57	4.78E‐23	
10702‐1_3	Q2UY09	COL28A1	Collagen type XXVIII alpha 1 chain	0.56	0.53	5.00E‐23	
3485‐28_2	P61769	B2M^a^	Beta‐2 microglobulin	0.53	0.51	1.56E‐22	This gene encodes a serum protein found in association with the major histocompatibility complex class I heavy chain on the surface of nearly all nucleated cells. The protein has a beta‐pleated sheet structure that can form amyloid fibrils in some conditions
11178‐21_3	Q4LDE5	SVEP1^+^		0.47	0.48	2.66E‐21	
6605‐17_3	P35858	IGFALS^+^	Insulin‐like growth factor‐binding protein acid‐labile subunit	1.67	1.56	9.10E‐21	A serum protein that binds insulin‐like growth factors, increasing their half‐life and their vascular localization. Production of the encoded protein, which contains twenty leucine‐rich repeats, is stimulated by growth hormone
2944‐66_2	P41271	NBL1^a^	Neuroblastoma 1, DAN family BMP antagonist	0.53	0.51	1.67E‐20	This gene product is the founding member of the evolutionarily conserved CAN family of proteins, which contain a domain resembling the CTCK (motif found in a number of signaling molecules. These proteins are secreted and act as BMP (bone morphogenetic protein) antagonists by binding to BMPs and preventing them from interacting with their receptors. They may thus play an important role during growth and development
8464‐31_3	Q2I0M5	RSPO4^a^	R‐spondin 4	0.69	0.67	2.04E‐20	This gene encodes a member of the R‐spondin family of proteins that share a common domain organization consisting of a signal peptide, cysteine‐rich/furin‐like domain, thrombospondin domain, and a C‐terminal basic region. The encoded protein may be involved in activation of Wnt/beta‐catenin signaling pathways
2609‐59_2	P01034	CST3^a^	Cystatin C	0.62	0.59	2.18E‐20	The cystatin superfamily encompasses proteins that contain multiple cystatin‐like sequences. This gene is located in the cystatin locus and encodes the most abundant extracellular inhibitor of cysteine proteases, which is found in high concentrations in biological fluids in all organs of the body
3024‐18_2	P08697	SERPINF2^a^	Serpin family F member 2	1.43	1.43	2.80E‐20	This gene encodes a member of the serpin family of serine protease inhibitors. The protein is a major inhibitor of plasmin, which degrades fibrin and various other proteins. Consequently, the proper function of this gene has a major role in regulating the blood clotting pathway
7211‐2_3	P07998	RNASE1	Ribonuclease A family member 1, pancreatic	0.36	0.33	3.58E‐20	This gene encodes a member of the pancreatic type of secretory ribonucleases, a subset of the ribonuclease A superfamily. The encoded protein is monomeric and more commonly acts to degrade ds‐RNA over ss‐RNA.
11388‐75_3	Q14508	WFDC2	WAP four‐disulfide core domain 2	0.56	0.52	5.01E‐20	This gene encodes a protein that is a member of the WFDC domain family and functions as a protease inhibitor in many family members. This gene is expressed in pulmonary epithelial cells
8368‐102_3	P20333	TNFRSF1B	TNF receptor superfamily member 1B	0.59	0.57	7.65E‐20	The protein encoded by this gene is a member of the TNF receptor superfamily. This protein and TNF receptor 1 form a heterocomplex that mediates the recruitment of two anti‐apoptotic proteins, c‐IAP1 and c‐IAP2, which possess E3 ubiquitin ligase activity. Knockout studies in mice also suggest a role of this protein in protecting neurons from apoptosis by stimulating antioxidative pathways
8469‐41_3	P18065	IGFBP2^a^		0.50	0.50	1.41E‐19	
13118‐5_3	Q9H4F8	SMOC1^a^	SPARC related modular calcium binding 1	0.72	0.72	2.05E‐19	This gene encodes a multi‐domain secreted protein that may have a critical role in ocular and limb development
13392‐13_3	P05026	ATP1B1	ATPase Na+/K+ transporting subunit beta 1	1.21	1.21	3.72E‐19	The protein encoded by this gene belongs to the family of Na+/K+ and H+/K+ ATPases beta chain proteins, and to the subfamily of Na+/K+ ‐ATPases. Na+/K+ ‐ATPase is an integral membrane protein responsible for establishing and maintaining the electrochemical gradients of Na and K ions across the plasma membrane
6392‐7_3	O76076	WISP2	WNT1‐inducible signaling pathway protein 2	0.59	0.56	7.90E‐19	A member of the WNT1 inducible signaling pathway (WISP) protein subfamily, which belongs to the connective tissue growth factor family. WNT1 is a member of a family of cysteine‐rich, glycosylated signaling proteins that mediate diverse developmental processes. It is expressed at high levels in bone tissue and may play an important role in modulating bone turnover
14088‐38_3	P24592	IGFBP6	Insulin‐like growth factor‐binding protein 6	0.70	0.66	1.02E‐18	
5496‐49_3	Q9HCB6	SPON1	Spondin 1	0.64	0.60	2.06E‐18	
6546‐41_3	Q9UBU2	DKK2	Dickkopf WNT signaling pathway inhibitor 2	0.58	0.55	2.64E‐18	
11196‐31_3	P12111	COL6A3	Collagen type VI alpha 3 chain	0.74	0.72	3.03E‐18	This gene encodes a protein that is a member of the dickkopf family. The secreted protein contains two cysteine‐rich regions and is involved in embryonic development through its interactions with the Wnt signaling pathway
3438‐10_2	O95633	FSTL3^a^	Follistatin‐like 3	0.61	0.57	4.30E‐18	Follistatin‐like 3 is a secreted glycoprotein of the follistatin‐module‐protein family

Note

A + denotes a protein that was replicated in the mass spectrometry validation.

FC cont to cent: Fold change comparing protein abundance in controls versus centenarians. Note that FC cont to cent >1 indicates a protein that decreases in centenarians, while FC cont to cent <1 indicates a protein that increases in centenarians.

FC off to cent: Fold change comparing protein abundance in centenarians' offspring versus centenarians. FC off to cent >1 indicates a protein that decreases in centenarians, while FC off to cent <1 indicates a protein that increases in centenarians.

P F test: *p*‐value from ANOVA *F* test to compare the three groups (centenarians, centenarians' offspring and controls), adjusted for sex and serum storage time.

^a^ A protein that was replicated in at least one independent study.

#### 2.1.2 Validation with mass spectrometry

We used high‐performance tandem mass spectrometry (MS/MS) with 10‐plex tandem mass tags (TMT) to profile in triplicates 10 of the 226 serum samples that were included in the original SomaScan experiment. The samples were selected uniformly from the age range 50–100 years. The MS analysis detected 675 proteins with at least one measured peptide and included 443 proteins in the SomaLogic array, 186 of which were in our aging signature. We analyzed the MS data using a Bayesian hierarchical model to detect proteins that correlated with age and identified 15 proteins of the aging signature that were significantly associated with age in the MS experiment (*p* < 0.05), and 12 protein of the aging signature that were more borderline in the MS experiment (*p*‐value between 0.05 and 0.10) with consistent effect (Table S1d). We only found one protein with statistically significant correlation with age that showed an effect opposite to that based on the SomaScan technology. The number of proteins with concordant and significant effects increased to 33 when we considered the overlap with those that correlated with age at 5% FDR in the SomaScan experiment. The set of validated proteins included IGFALS and SVEP1 and well‐established protein biomarkers of aging such as sex hormone‐binding protein (SHBG). Of the 186 proteins included in the aging signature, 129 (69%) had concordant effects, while only 128 of the 257 not in the aging signature (49%) were concordant. The concordance of the aging‐related proteins was highly significant (*p* from Fisher exact test 4E‐5).

#### 2.1.3 Functional annotation of the centenarian signature

Annotation of the results using the 50 Hallmark pathways (Liberzon et al., 2015) (http://software.broadinstitute.org/gsea/msigdb/genesets.jsp?collection=H) identified 12 pathways important to the immune system and the cell cycle with nominal level of statistical significance (20% FDR: Figure 1b). The complete set of results is in Table S2.

#### 2.1.4 Pathway projection analysis

Pathway projection analysis of all proteins in the SomaScan array identified 18 of 50 Hallmark pathways as significantly different between centenarians and the younger groups at 5% FDR (Figure 1a, right). Consistent with growing evidence of the role of peroxisomes in aging and cell senescence (Cipolla & Lodhi, 2017; Giordano & Terlecky, 2012), proteins encoding components of peroxisomes were the most significant set (*p* = 2E‐09) and were under‐expressed in centenarians' serum compared with younger individuals. Both the bile acid metabolism pathway (*p* = 4.4E‐06) and cholesterol homeostasis pathway (*p* = 0.0003) were also under‐expressed in centenarians, as well as proteins downregulated by KRAS activation (*p* = 0.0003), and proteins upregulated by TGFB1 (*p* = 7.2E‐07). Significantly over‐expressed sets of proteins in centenarians' serum included those upregulated in response to interferon‐gamma (*p* = 1.0E‐07), those involved in DNA repair (*p* = 7.0E‐05), MYC targets (*p* = 0.009), and those activated by P13K, ATK, and MTOR signaling (*p* = 0.02). Several of these pathways were also seen to be regulated on a gene level in preclinical models (Shavlakadze et al., 2019), including upregulation of Myc target genes (Shavlakadze et al., 2018). These results are consistent with well‐known Hallmarks of aging that include deregulated nutrient sensing, cellular senescence, and inflammation (Joseph et al., 2019; Singh et al., 2019). The full list of results is described in Table S3a. Two hundred and one specific gene sets from the “canonical pathways” compendium (Liberzon et al., 2011) were also significantly different in centenarians' serum compared with younger individuals (FDR < 0.1%) and included proteins involved in nicotinate and nicotinamide metabolism (up in centenarians' serum), and the IL‐6 signaling pathway (up in centenarians' serum). The complete set of results is in Table S3b.

### 2.2 Replication and characterization of the centenarian signature

We compared the list of significant proteins in four independent datasets.

#### 2.2.1 Replication with Spanish centenarians proteomics study

Santos‐Lozano and colleagues described a signature of 49 plasma proteins that differed between nine healthy centenarians and nine controls at 5% FDR (Santos‐Lozano et al., 2020). The protein signature was measured using tandem mass spectrometry with isobaric tags (TMT 10‐plex) and includes 28 proteins profiled in the SomaScan array, 17 of which were included in the aging signature and 13 (76%) of these proteins had concordant effects (Table S4a). Nine of the 28 proteins were not associated with being centenarians in our study (FDR > 10%). Interestingly, the set of 13 replicated proteins included IGFALS.

#### 2.2.2 Comparison with TwinsUK aging study

Menni et al. (2015) described a protein scan using plasma samples of 202 women from the TwinsUK study (mean age 65.3 (6.92)), and replication in plasma samples of 677 unrelated individuals from the AddNeuroMed, Alzheimer's Research UK, and Dementia Case Registry cohorts (mean age 76.96 (7.06)). The plasma protein profiles were generated using the SomaScan v3 that measured 1129 proteins in the samples from the TwinsUK study and using the SomaScan v2 that measured 1001 proteins in the independent replication set. Thirteen proteins were positively associated with chronological age in the TwinsUK study and eleven replicated in the independent set (Table S4b). Twelve of these proteins were present in the centenarian signature with consistent effects (over‐expressed in centenarians) and ranked between position 3 (PTN) and position 656 (ADAMTS5). Metalloproteinase inhibitor 1 (TIMP1) did not exhibit any variation in the NECS serum samples.

#### 2.2.3 Comparison with the Baltimore Longitudinal Study on Aging (BLSA)–GESTALT study

**FIGURE 2:**
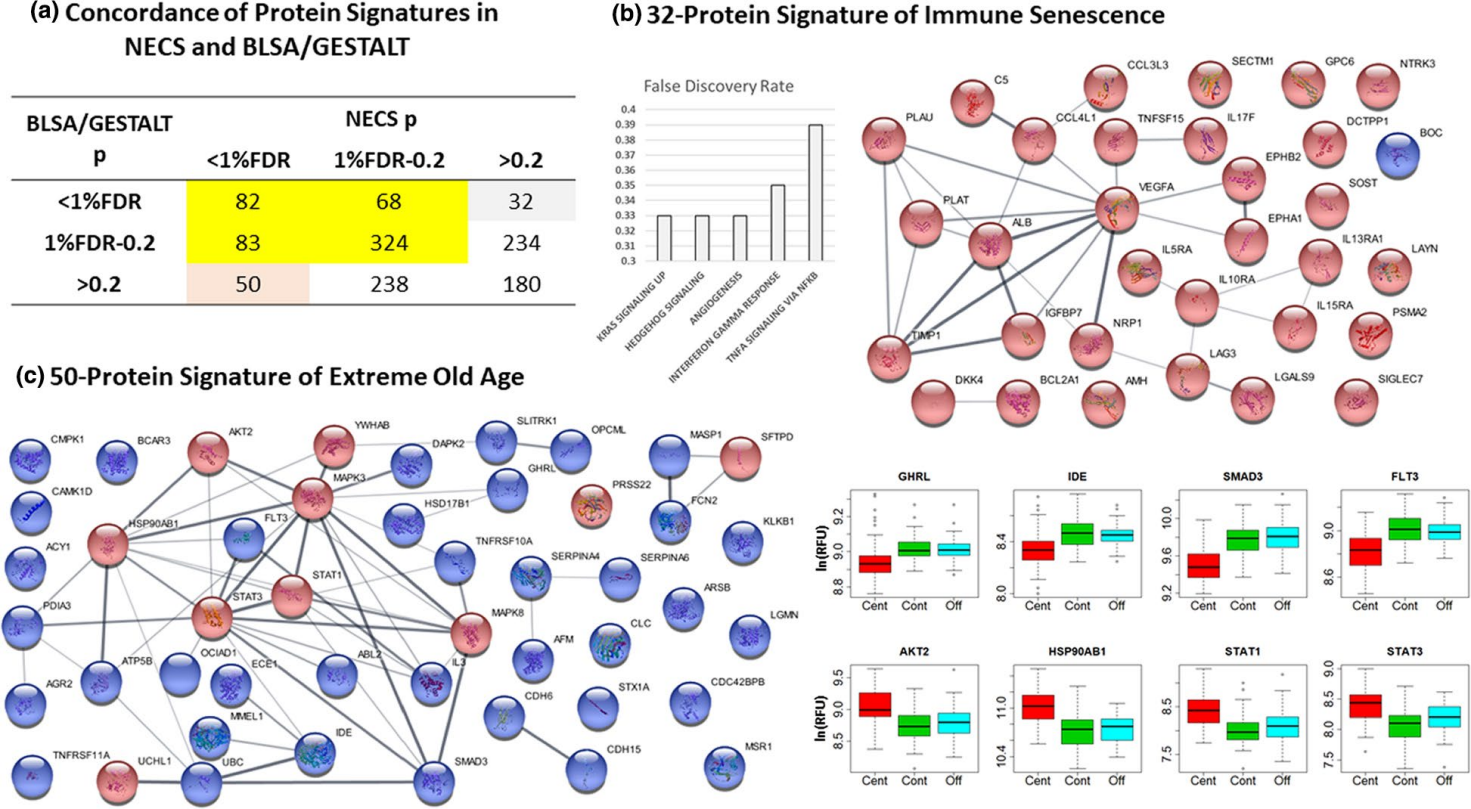
Comparison with proteomic profiles in plasma samples of BLSA/GESTALT participants. (a) Concordance table of the results for the 1291 aptamers common to the NECS and BLSA/GESTALT studies. The aptamers were grouped by level of significance (significant: *p* < 1% FDR), ambiguous (*p* between 1% FDR and 0.2), or not significant (*p* > 0.2). The concordance of the 82 significant results was highly significant (*p* from Fisher exact test <2.2e‐16). (b) Annotation of the 32 protein signature of immune senescence and their network of connection. The network was built using STRING (https://string‐db.org/), and nodes represent proteins (red denotes proteins that increase with age and blue denotes proteins that decrease with age) while edges are connections based on known interactions (magenta and cyan), text mining (light green), and co‐expression (black). (c) Network of connections between the 50 proteins of the extreme old‐age signature (red nodes = proteins over‐expressed in centenarians; blue nodes = proteins under‐expressed in centenarians), and selection of eight proteins that generate differential signal intensities in centenarians (red boxplots), compared with controls (green), and centenarian offspring (cyan). Values on the *y*‐axis are log‐transformed RFU

Tanaka et al. (2018) measured plasma protein profiles in 240 healthy men and women, ages ranging between 22 and 93 years, using the SomaScan 1.3K with 1322 aptamers, and discovered an aging signature of 217 proteins with 5% Bonferroni‐corrected level of significance. We identified 1291 aptamers present in both the platforms used to profile the NECS serum samples and the BLSA/GESTALT plasma samples, and compared the list of aptamers that correlated with age and extreme old age (being centenarian) using a 1% FDR. The cross‐classification table in panel a of Figure 2a shows that 82 aptamers were significantly associated in both analyses at 1% FDR, and the concordance of the 82 significant results was highly significant (Fisher's exact test *p* < 2.2e‐16). The table also shows that the associations of 180 aptamers were not significant in either analysis (*p* for age‐association in both analyses >0.2). Since the centenarian signature includes proteins that may be specific to centenarians serum, we investigated closely the set of 50 proteins that were significantly associated with being centenarian (<1% FDR) but were clearly not significantly associated with age in the BLSA/GESTALT study (*p* > 0.2), and the set of 32 proteins that were significantly associated with age in the BLSA/GESTALT study (<1% FDR) but were not significantly associated with being centenarians (*p* > 0.2).

Table S4c lists the set of the 32 proteins that include inhibitors of the WNT signaling pathway such as serum sclerostin (SOST) and “dickkopf WNT signaling pathway inhibitor 4” (DKK4) (Modder et al., 2011; Tanaka et al., 2018). These 32 proteins also include 14 involved in the immune system such as cytokines receptors (IL5RA, IL10RA, IL15RA, IL17F) and chemokines ligands (CCL3L1, CCL4L1), and their lack of difference in centenarians suggests a status of immune exhaustion or *immune senescence* (Fulop et al., 2018). Annotation using the 50 Hallmark pathways suggests that this set of 32 proteins is significantly enriched for proteins that are usually activated by KRAS (EPHB2, IL10RA, NRP1, PLAT, PLAU), and in the HEDGEHOG signaling pathway (NRP1, VEGFA), and they are downregulated in centenarians. The network in Figure 2, panel b, depicts the known relations between these proteins.

Table S4d lists the 50 proteins that are significantly different in centenarians' serum compared with the younger groups (<1% FDR) but are not correlated with age in the BLSA/GESTALT study (*p* > 0.2). This list includes 18 proteins in the top 10% of the centenarian signature—an almost fourfold enrichment compared with a random selection. The list comprises 24 glycoproteins and 18 immune system proteins including SMAD3, a critical modulator of many pathways that is under‐expressed in centenarians' serum, and STAT1 and STAT3, regulators of inflammatory response that are over‐expressed in centenarians' serum. The set also includes the following proteins that are under‐expressed in centenarians' serum: upstream regulators of MTOR (FLT3), GHLR which is an appetite‐regulating hormone, and the insulin‐degrading enzyme IDE that may play a role in the degradation and clearance of naturally secreted amyloid beta‐protein by neurons and microglia. Many of these proteins are known to interact (Figure 2c) and provide a signature of *extreme old age* characterized by increased inflammation and failing of vital biological processes.

We also conducted a meta‐analysis of the 557 aptamers that reached a *p* < 0.2 in both analyses producing 234 aptamers (targeting 232 proteins) that were associated with age at 1% FDR (Table S4e). Compared with the list of 217 published in Tanaka et al. (2018), the meta‐analysis adds novel protein biomarkers of aging including a significant negative association of growth hormone receptor (GHR) levels (*p* = 3.2E‐16) and of myostatin (MSTN) levels (*p* = 2.6E‐16) with age.

#### 2.2.4 Comparison with the INTERVAL study

Sun et al. (2018) described the association between age and 2327 aptamers mapping to 2143 protein measured with an expanded SomaScan array in the plasma of 3301 participants of the INTERVAL study (mean age approximately 44 years, *SD* = 4 years). Protein levels were rank‐inverse normalized, so the age effects reported in their analysis are not comparable with the age effects in our work or Tanaka's work (Tanaka et al., 2018). However, we were able to compare the concordance of the direction of effects. We identified 2317 aptamers common to the NECS and the INTERVAL study and removed aptamers with inconsistent age effects reported in Ref. (Sun et al., 2018) and Ref. (Tanaka et al., 2018), as well as aptamers not significantly associated with age in the INTERVAL study that are not interpretable because of the younger ages of the participants. These steps are summarized in Figure S4, panel a, and left 1096 aptamers associated with age in the INTERVAL study at 1% FDR. This set includes 296 proteins that do not change in centenarians' serum compared with younger people (Table S5a) and includes 17 of the 32 in the immune senescence signature shown in Figure 2b. Of 453 aptamers that reached a 1% FDR significance association with age in both studies (*p* from Fisher's exact test 1.471e‐10), 347 had concordant effects and are listed in Table S5b. The Venn diagram in Figure S4 shows that, between the lists validated in the INTERVAL study and the BLSA/GESTALT study, there is an overlap of 79, and 268 aptamers of the aging signature are replicated in the INTERVAL study bringing the number of aptamers from the aging signature with replication in at least one independent study to 502 (484 unique proteins). The list of these 502 aptamers is in Table S5c.

### 2.3 Survival signatures

The comparison of the centenarian signature with four independent studies of aging showed that many proteins of aging are highly expressed in centenarian serum in addition to protein signatures that are unique in centenarians. However, these cross‐sectional data do not capture the patterns of protein changes that occurred in the serum profiles of centenarians when they were much younger and that may have determined their extreme longevity. We therefore searched for proteins that correlate with longer survival in centenarians and, separately, in centenarians' offspring and control. We conducted the analyses separately in centenarians and in centenarians' offspring and controls because their mortality risk is very different.

#### 2.3.1 Signature of survival in centenarians

**FIGURE 3:**
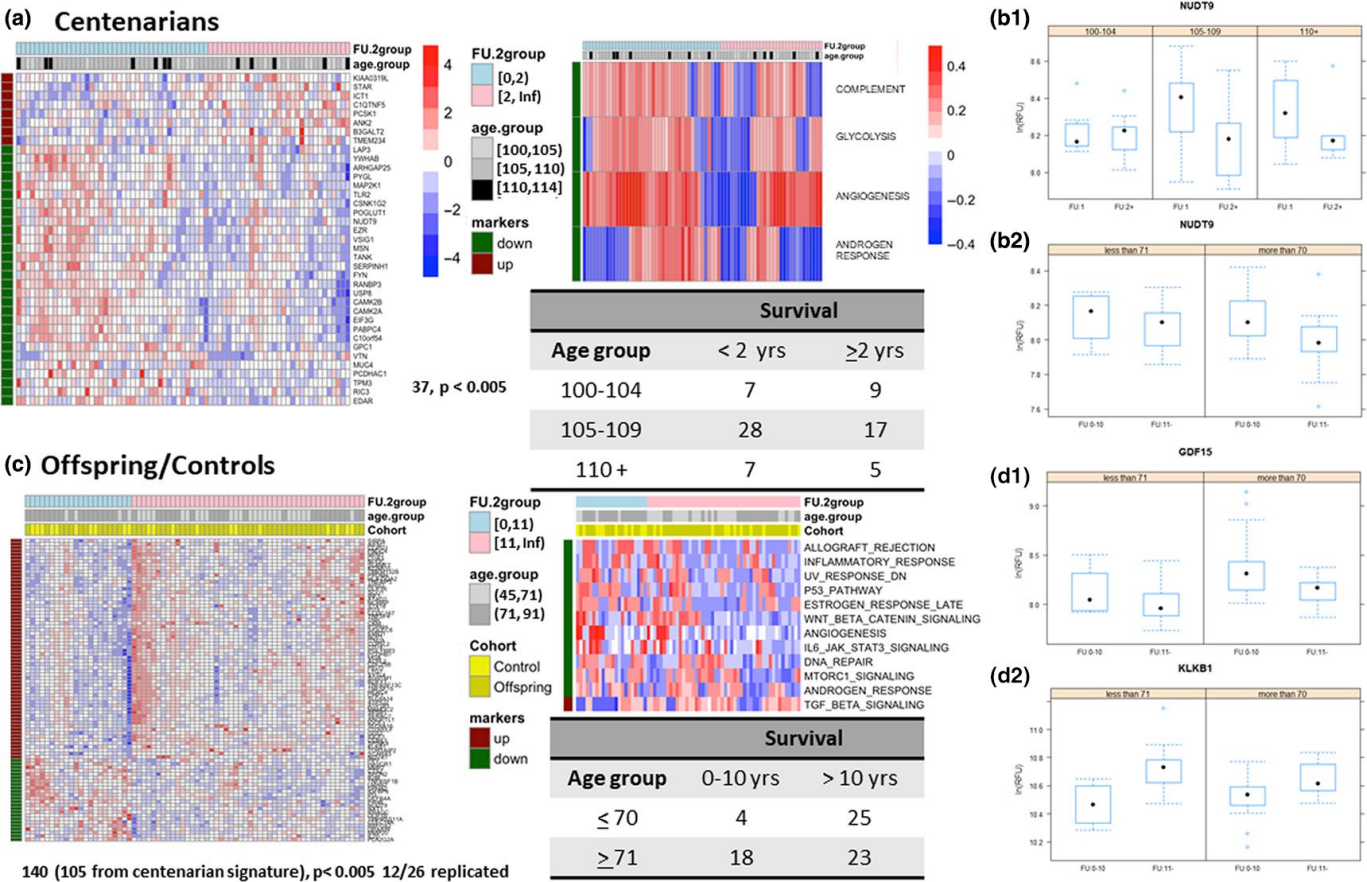
Longevity and extreme longevity signatures. (a) Heatmap of the 37‐protein signature in centenarians (nominal *p*‐value <0.005) and of the 4 Hallmark pathways that differentiate between shorter and longer survival in centenarians (*p*‐value <0.05). See the legend of Figure 1 for details of the heatmaps. (b.1) Conditional boxplots of ADP‐ribose diphosphatase (gene *NUDT9*) in centenarians. Each panel displays boxplots of log‐transformed RFU by survival (FU:1 = less than 2 years, FU:2+ =2 or more years). Different panels show increasing age at blood draw. (b.2). Conditional boxplots of ADP‐ribose diphosphatase (gene *NUDT9*) in centenarians' offspring and controls. The left panel displays boxplots of log‐transformed RFU by survival (FU:10 = up to 10 years, FU:11+ = 11 or more years), in subjects aged <71 years at blood draw. The right panel represents subjects aged 71 and older at blood draw. Both conditional plots show that ADP‐ribose diphosphatase (gene *NUDT9*) increases with older age but is lower in people with longer survival. (c) Heatmap of the 140‐protein signature (*p*‐value <0.005) and of the 12 Hallmark pathways that correlate with longer survival in longevity in centenarians' offspring and controls. (d1, d2) Conditional boxplots of log‐transformed RFU for GDF15 and KLKB1 in centenarians' offspring and controls stratified by survival (FU:10 = up to 10 years, FU:11+ = 11 or more years) and age at blood draw (<71 years or 71 and older). The plots show that GDF15 increases with age and lower levels are associated with longevity in centenarians offspring and controls, and KLKB1 expression decreases with age and higher values are predictive of longer survival

To represent longer than expected survival in centenarians, we chose 2 years which is the expected years of survival of a 100 year old born in 1900, and we stratified the set of 73 centenarians into 42 who survived less than 2 years after the blood draw and 31 who survived 2 years or more. We then correlated the protein data with these two groups, adjusting for sex and age at blood draw. Although no single aptamer reached a significant association with survival after correction for multiple testing, a set of 37 proteins were significantly associated with survival group with *p*‐value <0.005, which represents a 50% increase over the number expected by chance (Figure 3a, Table S6a). This set included 11 proteins that are also part of the centenarian signature and that increased in centenarians compared with younger individuals, but decreased with longer survival compared with shorter survival (see, e.g., Nudix hydrolase 9: NUDT9 in Figure 3). The exception to this pattern was PCSK1 that increased in centenarians and also increased with longer survival (Figure S5), suggesting that high expression of PCSK1 may promote extreme longevity. Pathway projection analysis using the 50 Hallmark pathways suggests a lower level of androgen response is associated with longer survival (*p* = 0.003, 13% FDR), as well as low levels of the complement pathway, glycolysis, and angiogenesis, although these three pathways reached only nominal significance (Table S6b).

#### 2.3.2 Signature of survival in centenarians' offspring and controls

We repeated a similar analysis in 70 centenarians' offspring and controls for whom we had information about death within or beyond 10 years from the time of the blood draw. We choose 10 years to represent longer than expected survival (longevity) since this is approximately the average number of years of life expected in individuals aged 70 years and born in the 1930 cohort. We stratified the 70 centenarians' offspring and controls into a group of 22 who died within 10 years from the blood draw and a group of 48 who survived beyond 10 years. We then correlated the protein data with these two groups, adjusting for sex, age at blood draw, and participant type. The analysis detected a set of 140 aptamers that were associated with survival (*p* < 0.005, 17% FDR) (Table S7, Figure 3c), including 115 that were also part of the centenarian signature. In particular, the signal of 86 proteins decreased in centenarians and increased with longer survival, while the signal of 24 proteins increased in centenarians and decreased with longer survival. Figure 3d shows two examples of the relevant proteins: GDF15 increased with older age but lower values were associated with longer survival, while kallikrein B1 (KLKB1) decreased with older age and higher values were associated with longer survival. Point variants of KLKB1 have been shown to associate with amyloid beta levels and thus atherosclerosis (Simino et al., 2017). We replicated the association with survival of 26 of these 140 markers in the InCHIANTI study using approximately 15 years of follow‐up data and 504 deaths. Leveraging the larger sample size of InCHIANTI, the association with survival was analyzed using the Cox‐proportional regression analysis, adjusted by age and sex, and 12 of these 26 proteins showed significant association with survival, with consistent effects in the two studies (Table 3).

**TABLE 3:** Replicated proteins that correlate with longer survival

SomaScan ID	UniProt	Gene symbol	NECS		InCHIANTI		
			FC long vs short survival^a^	*p* ^b^	log(HR)^c^	*SE*	*p* ^d^
4982‐54_1	P19957	PI3	0.72	4.46E‐05	0.77	0.11	1.11E‐11
4152‐58_2	P03952	KLKB1	1.14	0.000222	−0.87	0.22	6.74E‐05
2789‐26_2	P09237	MMP7	0.79	0.000393	0.51	0.10	7.13E‐07
3485‐28_2	P61769	B2M	0.84	0.000571	1.00	0.17	1.71E‐09
2670‐67_4	P06732	CKM	1.13	0.000969	−0.53	0.10	5.02E‐07
4834‐61_2	P29317	EPHA2	0.85	0.001597	1.17	0.19	6.52E‐10
2692‐74_2	P14555	PLA2G2A	0.75	0.001741	0.46	0.10	6.70E‐06
4374‐45_2	Q99988	GDF15	0.85	0.002899	1.03	0.12	1.11E‐16
5139‐32_3	O95185	UNC5C	0.86	0.003514	0.97	0.21	2.48E‐06
2609‐59_2	P01034	CST3	0.87	0.003525	1.16	0.22	1.22E‐07
3292‐75_1	P09326	CD48	0.89	0.004285	0.58	0.21	0.00438
5070‐76_3	O95407	TNFRSF6B	0.93	0.004857	0.36	0.13	0.004114

Note

A FC comparing long vs short survival lower than 1 means low abundance is associated with longer survival; hence, higher protein abundance is associated with higher mortality and a log(HR) >0. Similarly, a FC>1 means high abundance is associated with longer survival and hence higher protein abundance is associated with lower mortality and a log(HR) <0.

^a^ FC long vs short survival: fold change of protein abundance comparing individuals with survival >10 years versus less than or equal to 10 years.

^b^ *p* = *p*‐value from the t test to assess the significance of the fold change.

^c^ log(HR): log transformation of the hazard ratio for mortality in the InCHIANTI study.

^d^ *p* = *p*‐value from the t test to assess the significance of the hazard ratio.

When comparing the two signatures of survival identified in centenarians and in the younger group, only Nudix hydrolase 9 (NUDT9) was significantly associated in both and was also in the centenarian signature. Pathway projection analysis using the 50 Hallmark pathways detected 12 pathways associated with survival (Figure 3c). Upregulation of the TGF‐beta signaling pathway was associated with longer survival, while downregulation of all other 11 pathways was associated with longer survival. Six of the 12 pathways overlapped with those identified in the pathway projection‐based analysis of the aging signature in Figure 1a (right), and the association with aging and survival was in opposite directions. Five of these pathways (inflammatory response, UV response, P53 pathway, WNT/beta‐catenin signaling pathway, MTORC1 signaling, and androgen response) did not reach nominal significance in the aging analysis.

### 2.4 Centenarians and cellular senescence

We annotated the various signatures by their enrichment for senescence‐associated secretory phenotypes (SASP) using the SASP Atlas (www.saspatlas.com) described in Ref. (Basisty et al., 2019). Of the 4118 proteins linked to aptamers in the SomaLogic array, 417 overlapped with the list of 1140 SASP proteins that reached a significant differential expression at 5% FDR in human lung fibroblast (IMR90) or renal cortical cell treated with one of three senescence‐inducing stimuli: X‐ray irradiation, RAS overexpression, and the protease inhibitor atazanavir. The 1312‐protein centenarian signature included 174 SASP proteins (13%) that represents a significant enrichment (*p* = 8.584e‐06). The 32‐protein signature of immune senescence included seven SASP proteins (22%, *p* = 0.081), while the 50‐protein signature of extreme old age included eight SASP proteins (16%, *p* = 0.159) (Figure S5). The overall list of 484 aging‐associated proteins that replicated in at least one study included 84 SASP proteins (7.4%, *p* = 2.043e‐07). The 37‐protein survival signature in the oldest old included six SASP proteins (16.2%, *p* = 0.093), while the 140‐protein survival signature in the younger group included 14 SASP proteins (10.0%, *p* = 1) (Figure S5). These sets are summarized in Table S8.

### 2.5 Network analysis

**FIGURE 4:**
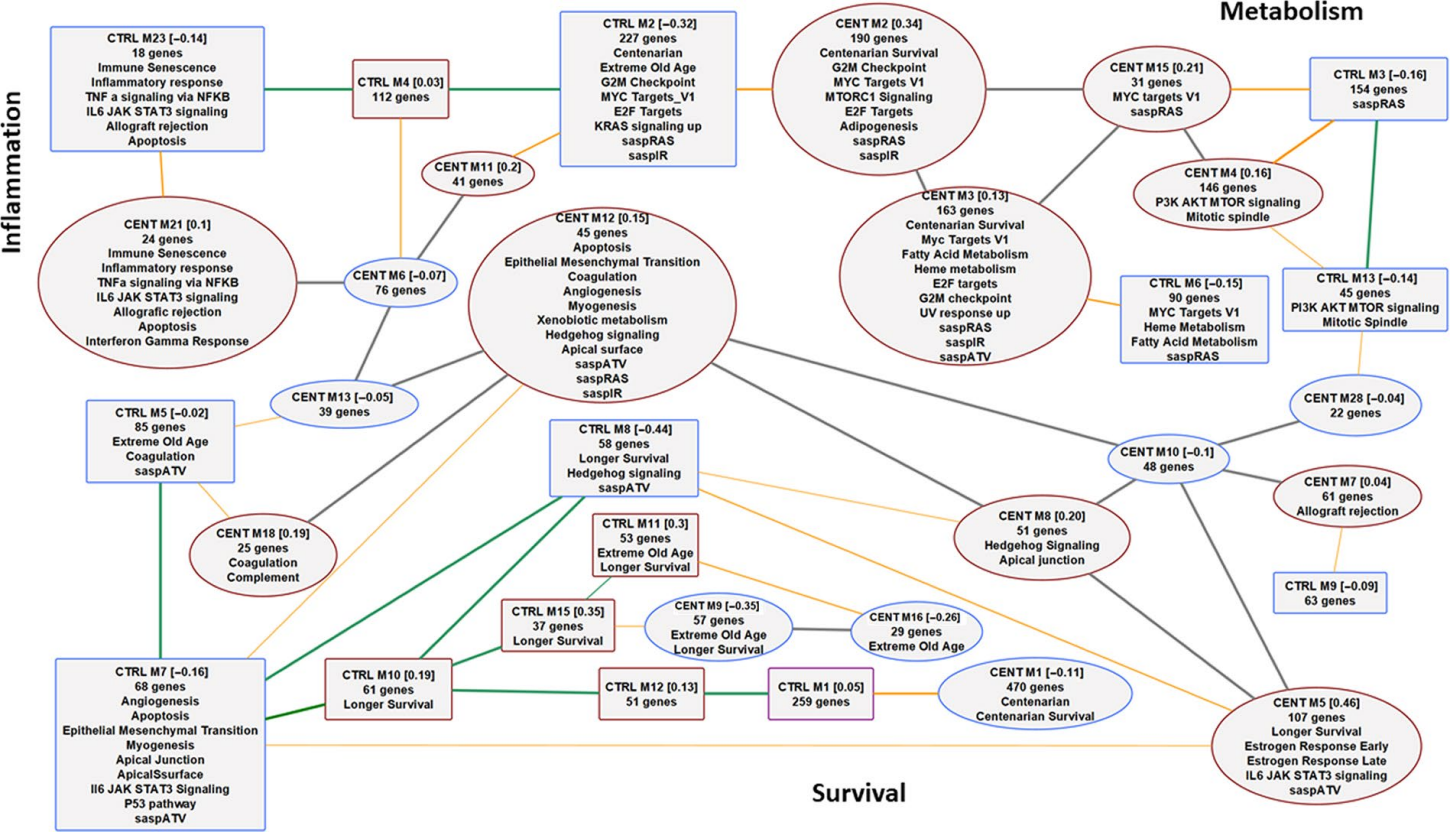
Overlap of modules of co‐regulated proteins in centenarians, and centenarians' offspring and controls. Squares denote protein modules in centenarians' offspring and controls (CTRL), and ellipses denote protein modules in centenarians (CENT). A red border denotes upregulated modules and a blue border denotes downregulated modules. Green edges represent correlations between centenarians' offspring and control modules, and gray edges represent correlation between centenarian modules. The numbers within square brackets represent the average standardized expression comparing CTRL to CENT in the CTRL modules and the other way round in CENT modules. For example, CENT M1 [−0.11] means that the average standardized expression of the proteins in the module comparing centenarians to offspring/control is −0.11. Gray edges represent correlation between eigengenes >0.8. Orange edges represent a >10% overlap based on Jaccard's concordance index. Each module was annotated with enrichment for the 50 Hallmark pathways, the 1312 centenarian signature, the 32‐protein signature of immune senescence, the 50‐protein signature of extreme old age, the 37‐protein signature of survival in the centenarians, the 140‐protein signature of survival in the offspring and controls, and the three SASP signatures of senescence

We generated co‐expression networks in the serum protein data of the centenarians and of the centenarians' offspring and controls, to test whether the two groups are characterized by different modules of correlated proteins that could point to additional biological mechanisms associated with older age and extreme longevity not detected by the previous analyses. The analysis identified 29 modules of co‐expressed proteins in centenarians (Table S9), with size ranging from 21 to 478 aptamers and median size 31 aptamers, and a large “null” module of 2813 aptamers (i.e., a module whose aptamers had no sufficiently high co‐expression with other groups of aptamers). Nineteen of the 29 modules were highly correlated with each other (17 gray edges between ellipse nodes in Figure 4. The same analysis in centenarians' offspring and controls generated only 23 modules of co‐expressed proteins with size ranging from 20 to 264 and median size 51, and a large null module of 2776 aptamers. Thirteen of the 23 modules were highly correlated with each other (11 green edges between rectangular nodes in Figure 4. We annotated the modules by their enrichment for the 50 Hallmark pathways, the new signatures discovered in the earlier analyses, and the senescence signatures determined by X‐ray irradiation, RAS overexpression, and protease inhibitor atazanavir (Basisty et al., 2019). We then identified modules in centenarians and in centenarian offspring and controls with a large number of overlapping aptamers, and connected them to emphasize their similarities (orange edges in Figure 4, Figure S6).

This analysis showed that the many modules discovered in centenarians have similar proteins to modules discovered in centenarians' offspring and controls but differ in abundance (orange edges, Figure 4). For example, module M1 in centenarians included 80% of the proteins in module M1 of centenarians' offspring and controls that were on average downregulated in centenarians. Module M3 in centenarians included 163 proteins that were more abundant in centenarians and overlapped with 80% of the proteins in module M6 of centenarians' offspring and controls. Both modules were enriched for Myc targets, pathways involved in metabolism, and SASP. Module M3 in centenarians was also enriched for proteins of the extreme longevity signature pointing to a connection between markers of longer survival in the extreme old, gene regulation, and metabolism. These conserved modules likely represent mechanisms that are important for aging and for longevity.

The analysis also highlighted differences in the structure of modules of co‐expressed proteins in the two groups. For example, module M2 in both groups shared the cell‐cycle component (E2F, G2M, Myc) and extreme old‐age signatures, but only the centenarians' module was enriched for mTORC1 and adipogenesis, and only the control module was enriched for KRAS (Figure 4). Other examples are discussed in Figures S7–S9. The fragmentation of modules of serum proteins in centenarians suggests a change of co‐regulation of genes involved in important biological processes of older age, including metabolism and inflammation. The analysis also discovered modules in the two groups of subjects that were enriched for the survival and extreme old‐age signatures, for example, modules M9 and M16 in centenarians, and modules M10, M11, and M15 in offspring and controls (Figure 4, Figure S9), pointing to these modules as potential new biological processes of longevity.

An overall summary of the results and annotation of each aptamer in the SomaScan array from the various analyses is in Table S10.

## 3 DISCUSSION

### 3.1 Overview of the results

The discovery of serum proteins as potential biomarkers of aging and disease has been challenging. However, the SomaScan technology has been shown to provide a sensitive and robust way to measure a large number of serum proteins with high reproducibility (Candia et al., 2017). With access to a SomaScan array of 4785 aptamers tagging 4116 unique proteins and serum samples from centenarians, centenarians' offspring, and unrelated controls age‐matched to the offspring but without familial longevity, we were able to discover a 1312‐protein signature that distinguishes centenarians' serum from that of old individuals (mean age 71 years). We validated 17 of these proteins using MS of 10 serum samples included in the SomaScan experiment. Comparison of our results with independent studies of aging produced a list of 484 proteins (502 aptamers) that were replicated in at least one study and include both novel and well‐established biomarkers of aging and cell senescence (e.g., GDF15, which is associated with anorexia and cachexia, phenotypes commonly observed in the elderly (Mullican & Rangwala, 2018)). Thus, the centenarian protein signature is enriched of aging proteins.

The most striking feature of the centenarian protein signature was the size: 1312 serum proteins (1428 aptamers) were different in centenarians with strong statistical significance. The discovery of a large number of significant proteins is likely due to our adoption of the largest SomaScan array used in a study of aging and longevity to date, to the high sensitivity of the technology (Gold et al., 2010), and to the inclusion of very old individuals that likely boosted our statistical power. Interestingly, median fold changes were moderate and, on average, proteins changed by 12%–25% comparing centenarians to their offspring and controls. Other studies of aging and longevity have shown that a large number of serum proteins change with age, and most of the changes are small (Lehallier et al., 2019). The 30 most significant aptamers were mostly increased in centenarians, but the overall signature included approximately the same number of over‐expressed and under‐expressed proteins in centenarians compared with younger individuals. The centenarian signature included well‐known biomarkers of aging (Justice et al., 2018) and was enriched for many Hallmark pathways pointing to inflammation, innate immunity, cell‐cycle regulation, and cancer‐related processes as important aging processes. The analysis was adjusted by sex, but we did not attempt separate analyses for the two sexes, and we expect that larger studies will be powered to discover sex‐specific aging signatures.

Our analysis identified about 100 proteins with a fold change of at least 1.5, but with this small set we did not detect any strongly significant pathway enrichment when we applied correction for multiple comparisons. It is of note that this smaller set was enriched for proteins involved with regulation of insulin‐like growth factor and metabolism of lipids at FDR ~20%.

The centenarian protein signature provided a list of proteins that were enriched for several biological processes. To directly test which biological processes were involved in differentiating centenarian serum from younger individuals, we also employed a pathway projection analysis in which we analyzed the differences in scores of known pathways among centenarians, centenarians' offspring and controls. This analysis showed that several well‐known aging pathways, for example, the mTOR pathway and the angiogenesis pathway (Ghaben & Scherer, 2019; Lopez‐Otin et al., 2013), increase their level of activity in centenarians, while few pathways reduce their level of activity in centenarians. Interestingly, targets of Myc and mTOR were the least significant Hallmarks; however, they did score as significant (Table S3b). In preclinical models, Myc hypomorphs (heterozygotic mice) were shown to live significantly longer than controls (Hofmann et al., 2015), and agents that inhibit mTORC1 signaling, such as rapamycin or other rapalogs, have been shown to increase lifespan and delay or prevent age‐related phenotypes (Kennedy & Lamming, 2016). Myc target genes have been shown to increase as a function of age in rats and are counter‐regulated by mTORC1 inhibition (Shavlakadze et al., 2018), and thus, the increase observed in centenarians is consistent with centenarians expressing normal age‐related changes, only much later than usual (Hofmann et al., 2015). Also, mTOR signaling itself has been shown to increase in muscle from older subjects (Joseph et al., 2019), giving a potential mechanism for the increase in Myc targets in humans, and further rationale for using mTORC1 inhibitors.

#### 3.1.1 Novel signatures of senescence and extreme old age

By studying the differences between the centenarian protein signature and proteins of aging in the BLSA/GESTALT study, we showed that centenarians carry a unique 50‐protein signature of “extreme old age,” and a 32‐protein signature of “immune exhaustion” that is consistent with immune senescence (Fulop et al., 2018). These signatures point to interesting processes linked to extreme old age.

The 32‐protein signature of immune senescence comprises aging proteins that did not change in centenarians compared with centenarians' offspring and controls. This signature included serum sclerostin (gene *SOST*) that has been reported to increase with older age (Modder et al., 2011; Tanaka et al., 2018), but the level in centenarians was undistinguishable from the younger groups. Higher serum levels of *sclerostin* have been linked to increased risk for fracture in aging individuals (Ardawi et al., 2012), and compounds that decrease *SOST* expression are targets for treatment of osteoporosis (Delgado‐Calle et al., 2017), so that the unchanged level seen in centenarians may describe a protective mechanism. *SOST* is an inhibitor of the WNT signaling pathway as dickkopf WNT signaling pathway inhibitor 4 (DKK4), which also increases with older age (Tanaka et al., 2018) but was unchanged in centenarians. The 32 proteins include several cytokines receptors (IL5RA, IL10RA, IL15RA, IL17F) and chemokines ligands (CCL3L1, CCL4L1) that increase with age, and their lower than expected levels in centenarians could suggest a status of immune exhaustion or immune senescence that may kick in to protect individuals at very old age (Fulop et al., 2018). This 32‐protein signature includes seven SASP proteins (Figure S5), including TMP metallopeptidase inhibitor 1 (TIMP1), and insulin‐like growth factor protein 7 (IGFBP7). Interestingly, suppression of TIMP1 in mice has been linked to maintenance and expansion of stem cells with no increased risk of cancer (Jackson et al., 2015), while the serum level of IGFBP7 is a marker of tissue aging and heart failure (Januzzi et al., 2018). Unchanged levels in centenarians compared with the younger age groups may indicate a slower aging rate, or good cardiac function. However, a note of caution is that blood collection procedures may affect the ability to detect some of these proteins in blood. This limitation is known for TIMP1 and other metalloproteinases (Jung et al., 2008; Losanoff et al., 2008; Mannello, 2008; Mannello & Jung, 2008; Mannello et al., 2008).

The 50‐protein signature of extreme old age included proteins that do not correlate with age but appear to be dysregulated in centenarians. This set includes eight SASP proteins, for example, 14‐3‐3 protein β/α (YWHAB) and HSP90AB1, which may play a role in apoptosis and inflammation and is a tissue‐specific biomarker of survival in ovarian cancer. The insulin‐degrading enzyme (IDE) plays a role in cellular breakdown of insulin, islet amyloid polypeptide (IAPP), and other peptides and may have a role in clearance of amyloid beta‐protein secreted by neurons. Ghrelin (GHR) induces the release of growth hormone from the pituitary gland and has an appetite‐stimulating effect that induces adiposity and stimulates gastric acid secretion. The levels of both IDE and GHR are down in centenarians, and this suggests failing of vital biological processes rather than expression of longevity markers. In addition, the decrease in GHR is consistent with the increase in GDF15—together predicting an anorexic phenotype. Thus, this particular signature is likely to be enriched in biomarkers of extreme old age that can inform about molecular functions toward the end of life but are unlikely to suggest the protective mechanisms that allowed centenarians to reach extreme old ages.

#### 3.1.2 Distinction between biomarkers of longevity and drivers of longevity

The macroscopic serum protein differences between centenarians and centenarians' offspring and controls likely gave large statistical power to discover many proteins. However, it is unlikely that all of these markers are “causal” or “drivers” of longevity, and a major challenge will be to distinguish the serum proteins that represent an effect of aging from proteins that represent processes that cause aging and extreme longevity. This distinction is important to identify targets for healthy aging therapeutics rather than simply biological markers of aging. The network analysis of serum protein in centenarians' offspring and controls showed that many serum proteins cluster in modules of co‐expression, suggesting that there may be a small number of driver proteins that co‐regulate downstream proteins. This result is consistent with the analysis in Emilsson et al. (2018) that showed a similar number of protein modules that are enriched for biological processes. The modules of proteins in centenarians were enriched for biological processes that are important for aging and longevity, and some protein modules were conserved in centenarians but others fragmented into smaller modules. For example, only one small protein module in offspring and controls was enriched for the mTOR signaling pathway, while two centenarians' modules were enriched for the mTOR and mTORC signaling pathways. This change in connectivity of groups of proteins suggests that extreme old age may affect gene regulation but larger datasets of protein profiles in centenarians' serum will be important to discover the processes that determine this loss of regulation. We expect that the integration of protein signatures with genetic signatures and other molecular data, including in particular methylation, will help with interpreting the patterns of conserved and fragmented modules and will point to important drivers of aging and extreme longevity. We also expect that larger samples of centenarian offspring and controls will reveal modules that are different between the two groups and point to earlier molecular changes that precede extreme longevity.

Consistent with previous work, the network analysis also suggests that serum proteins may provide insights about biological processes linked to aging. Emilsson et al. (2018) showed that indeed co‐expression networks revealed by studying serum proteins resemble those identified in many solid tissues, thus suggesting that serum can be used to infer general biological processes relevant to aging. In addition, parabiotic experiments have suggested that serum and plasma proteins may regulate complex biological processed linked to aging (Conboy et al., 2013).

#### 3.1.3 Signatures of survival

Our analysis identified a 140‐protein signature of survival in older adults and a 37‐protein signature of survival in centenarians. The level of statistical significance of the signature of survival in centenarians was weak (Table S6a, *p* < 0.005), and additional studies with larger sample sizes will be necessary to replicate and strengthen this result. The signature of survival in centenarians' offspring and controls was more robust and included 23 significant proteins at 10% FDR, 58 significant proteins at 11% FDR, and up to 140 proteins at 17% FDR (Table S7). Noticeably, 12 of these proteins were replicated in the InCHIANTI study using a more advanced statistical analysis. Many of the serum proteins that predict longer survival in centenarians' offspring and controls are also biomarkers of aging that appear to increase or decrease later in individuals who live longer. This result is consistent with the hypothesis that centenarians aged at a slower rate and maintained a healthy profile of these proteins as they aged (Ferrucci et al., 2020), but we do not have complete data yet to show how much younger than expected centenarians are, based on protein data.

In the absence of data on the serum of centenarians when they were younger, we analyzed the serum proteome of centenarians' offspring who tend to age more healthily than age‐matched controls without familial longevity (Terry et al., 2002). We identified 237 aptamers that were nominally differentially expressed between centenarians' offspring and controls (after adjusting for sex and sample age) but did not reach statistical significance after correction for multiple testing (Table S1e). Ninety‐six of these aptamers overlapped with the centenarian signature, and the signal of 59 aptamers (targeting 57 unique proteins) showed a potentially delayed aging effect in centenarian's offspring relative to controls. Specifically, six aptamers whose targets decreased with older age were higher in centenarians offspring compared with controls, and 53 aptamers whose targets increased with older age were lower in centenarians. However, larger sample sizes will be needed to reach statistical significance accounting for multiple testing. For example, we calculated that for the largest effect detected in this analysis to pass the Bonferroni‐corrected significance threshold we would need at least 250 samples, hence twice the current sample size. The Long Life Family Study (Newman et al., 2011) will generate a larger serum protein dataset in the next 2–3 years, and these data will enable us and others to conduct more in depth analyses. Larger samples of centenarians' offspring and control serum profiles will likely detect also differences in the network structures that we did not notice in our small set.

Our study also suggests that signatures of survival appear to be age‐dependent. In fact, the two signatures of survival in centenarians and in the younger age groups overlapped by only one protein, NUDT9, a member of a superfamily of enzymes involved in purine metabolism and in the regulation of Transient receptor potential cation channel subfamily M member 2 (gene *TRPM2*) (Wang et al., 2018). NUDT9 expression is reportedly localized to mitochondria (Perraud et al., 2003), and it will be interesting to determine whether it regulates mitochondrial function, which declines with age (Sun et al., 2016). In addition, five of the pathways involved with longer survival were not significant in the centenarian signature, thus suggesting that healthy aging is not only the result of slowing the aging rate.

### 3.2 Limitations

The data in this analysis include individuals with ages ranging from 45 years to 114 years at blood draw, but only a small number of study participants were younger than 60 years. Studies with a larger number of younger individuals and different technologies are needed to provide a more comprehensive assessment of the proteins that correlate with longevity. In addition, with the SomaScan array we could profile more than 4000 proteins, but this number is much smaller than the serum proteome. More comprehensive protein profiles will likely expand the set of proteins that regulate aging and longevity and possibly point to new important biological processes.

## 4 CONCLUSIONS

Analysis of centenarians, their offspring, and controls age‐matched to the offspring helped to demonstrate that centenarians express many of the protein signatures associated with aging, including common age‐related pathway changes such as interferon‐gamma signaling, mTOR signaling, the SASP signature, and Myc pathway upregulation. In addition, the patterns of some of these signatures in individuals with longer survival suggest that centenarians may have aged “slower” than other humans. What is of additional interest is that there are distinct patterns of proteins that are unique to centenarians. These findings suggest possible mechanisms that might be unique to the extreme aged, and are thus worth further exploration, as potential protective pathways that might be of use in treating age‐associated diseases.

## CONFLICT OF INTEREST

M.M. and L.L.J. are employees and stockholders of Novartis. D.J.G is an employee and stockholder of Regeneron Pharmaceuticals.

## AUTHORS' CONTRIBUTION

PS, DJG, LJ, MM, SM, and TTP contributed to study design, analysis plan, interpretation of the results, and drafting of the manuscript. PS, AG, AB, AF, MM, SM, and TT conducted the analyses. TT and LF conducted replication studies. KBC, GVD, and CEC contributed to the validation using MS. All authors contributed to the final version of the manuscript.

### OPEN RESEARCH BADGES

This article has earned an Open Materials Badge for making publicly available the digitally‐shareable data necessary to reproduce the reported results. The data is available at https://GitHub.com/montilab/ProteomicsSignaturesCentenarians

## Supporting information

Appendix S1

## Data Availability

Data are available on request due to privacy/ethical restrictions.
