# Supplementary material for: Protein signatures of centenarians and their offspring suggest centenarians age slower than other humans
Source: Aging Cell. 2021 Jan 29;20(2):e13290. doi: 10.1111/acel.13290 (PMC7884029; doi:10.1111/acel.13290)
Supplement: Supplementary file 1 — Appendix S1 [file ACEL-20-e13290-s001.zip › acel13290-sup-0001-FigS1-S9.pdf]

Supplement Figures

Supplement Figure 1

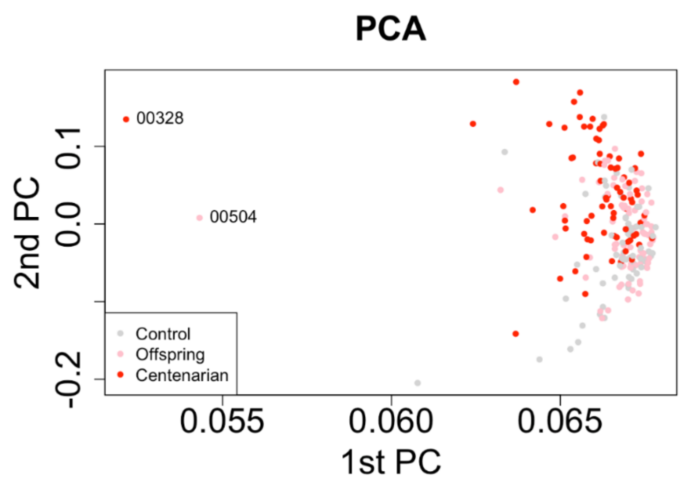

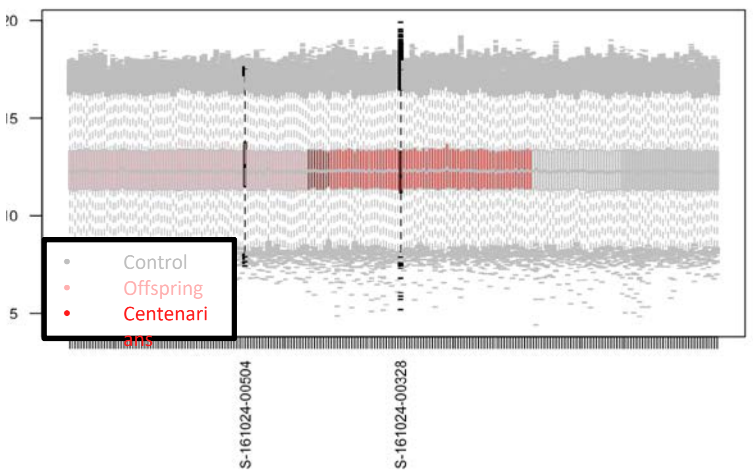

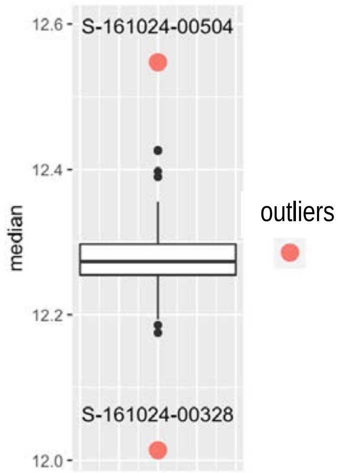

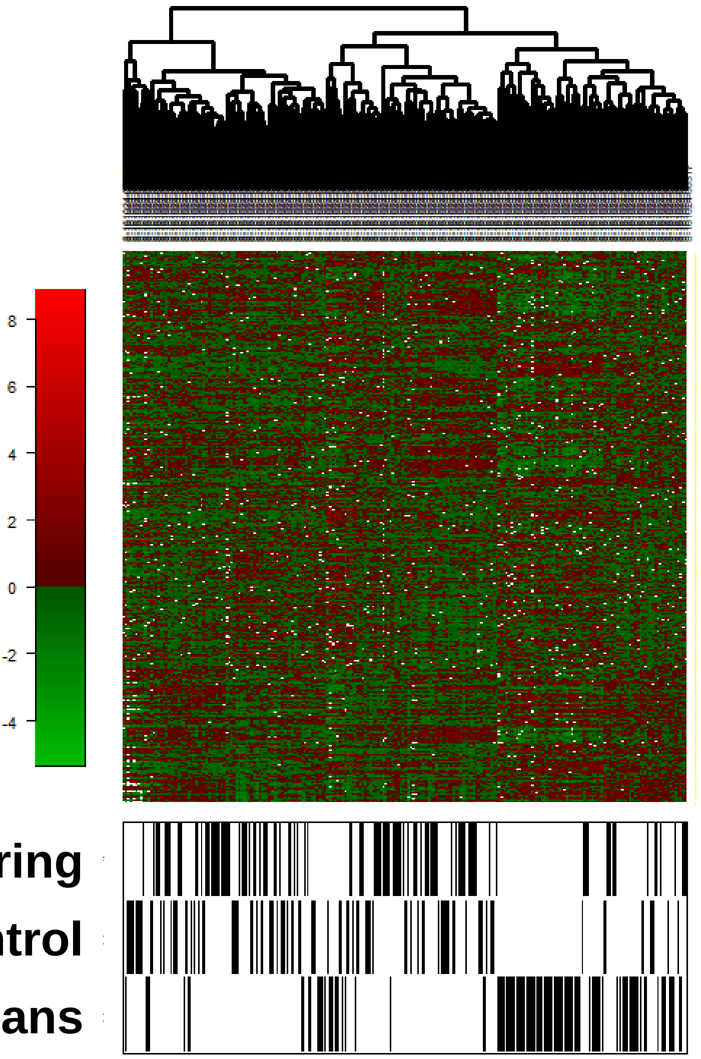

Principal component analysis left), side-by-side boxplots of log2 proteins RFU by samples middle) and boxplot of the median protein expression for each sample, with the two outlying samples in red. The first two principal components explained 94% of the total variability. The median expression of the two samples in red differed by more than the 1st quartile -1.5* the interquartile range, and the 3rd quartile + 1.5* the interquartile range were detected and the two samples were removed from the subsequent analyses.

The heatmap in the right panel shows the complete set of 224 samples and 4785 analytes that passed the quality control steps. The heatmap displays the normalized intensities by analytes (rows, sorted by hierarchical clustering) and samples (columns, sorted by hierarchical clustering). The “barcoding” at the bottom shows the 3 comparisons groups sorted by hierarchical clustering: centenarians cluster in a group that is different from the other 3 groups, suggesting that a large number of proteins may be linked to aging.

Supplement Figure 2

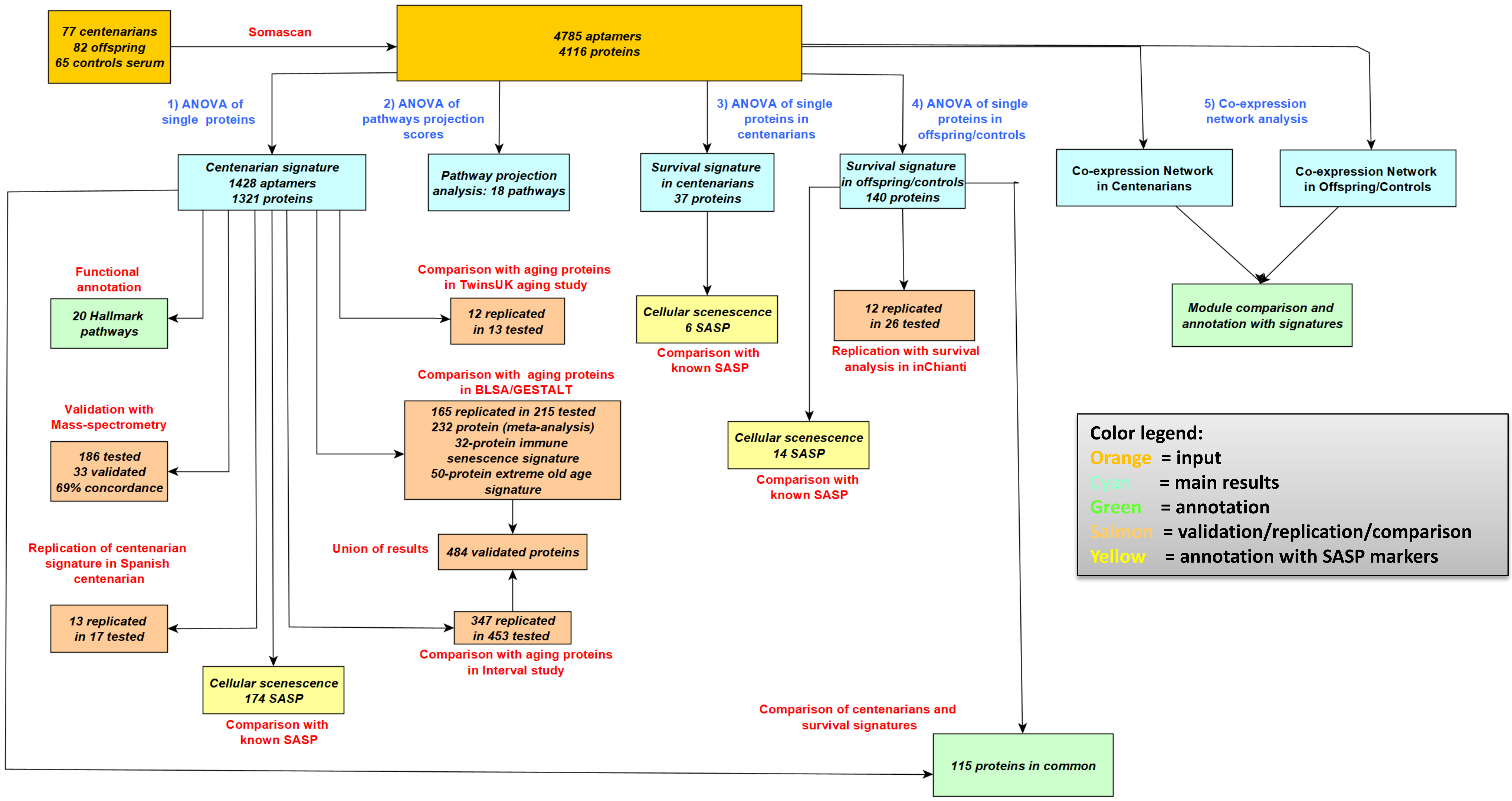

Supplement Figure 3

- The scatter plots show the agreement between estimates of age effects in the NECS and BLSA/GELSTALT studies. There are just few proteins that decrease with older age in the BLSA study. We see more negative changes with older age in NECS. The age effects in NECS are on average bigger, as shown by the plots above and below the diagonal.

a)

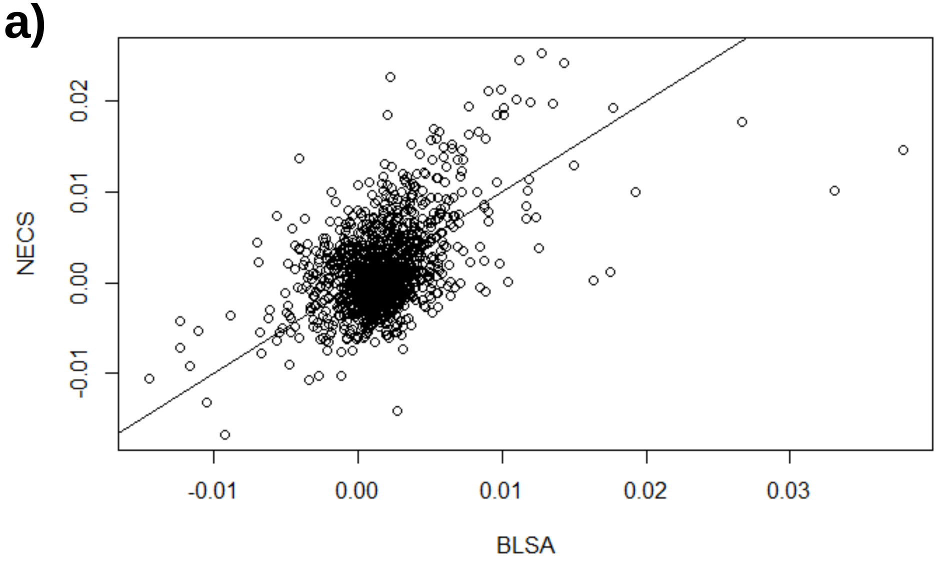

Scatter plot of age effects in BLSA and NECS studies, for all 1291 analytes measured in both studies.

b)

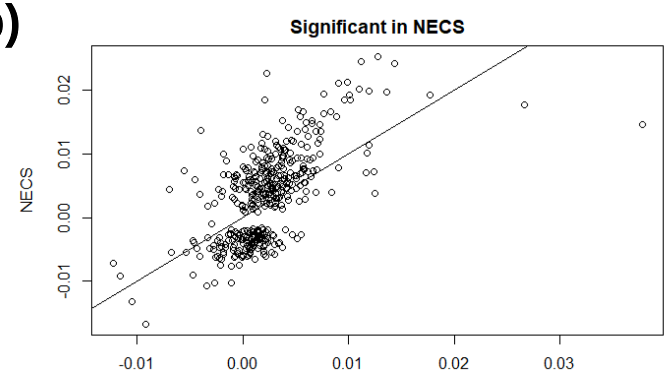

Scatter plot of age effects in BLSA and NECS studies for the subset of 408 proteins significant in NECS (1%FDR in 1291)

c)

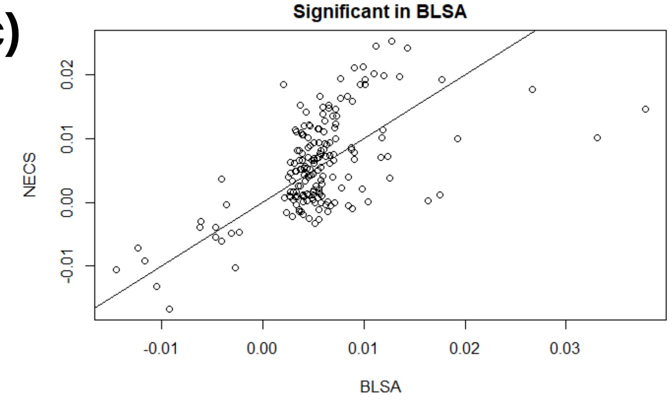

Scatter plot of age effects in BLSA and NECS studies for the subset of 102 proteins significant in BLSA (1% FDR in 1291).

Supplement Figure 4: Replication with Interval Study

a)

Interval p	NECS p <1% FDR	NECS p 1%FDR-0.2	NECS p >0.2
**<1% FDR**	453	313	296
**1%FDR-0.2**	214	217	238
**> 0.2**	160	192	234

Remove uncertain results in INTERVAL study ⇒

b)

Interval trend	NECS trend Cent up	NECS trend Cent flat	NECS trend Cent down
**Age up**	178	167	25
**Age down**	81	129	169

c)

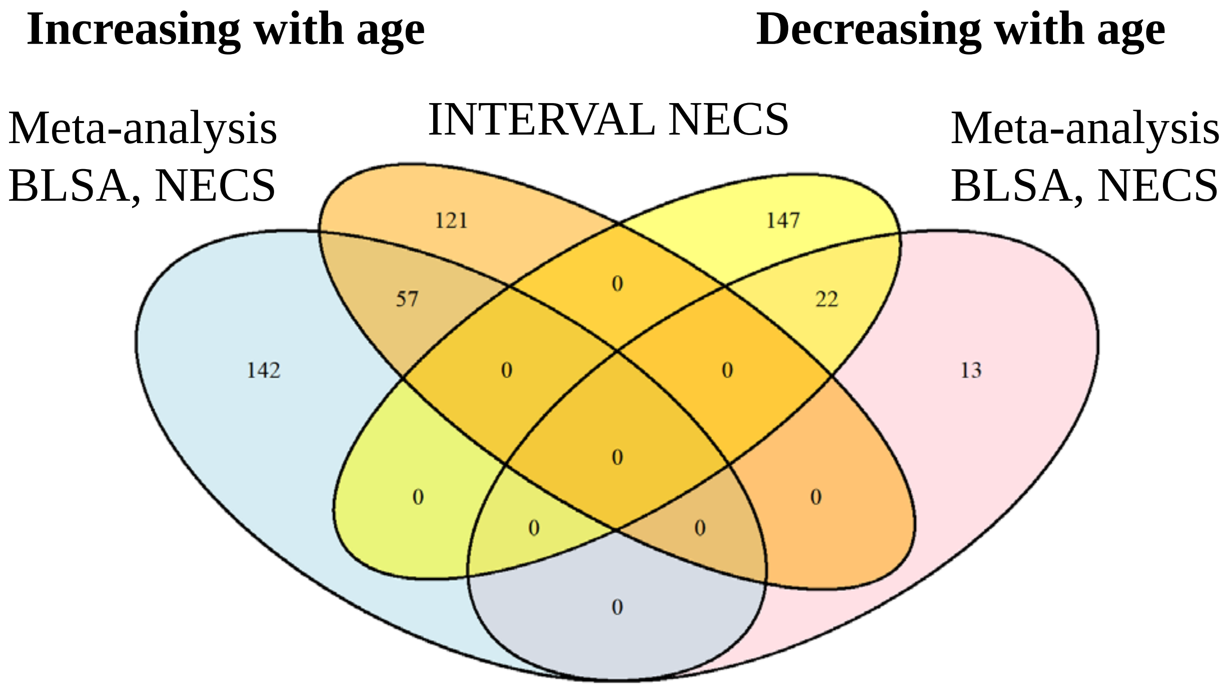

Robust list of 502 aptamers (484 proteins)

Supplement Figure 5: Overlap with SASPs

SASP proteins in the 32-protein signature of immune senescence and their secretion level

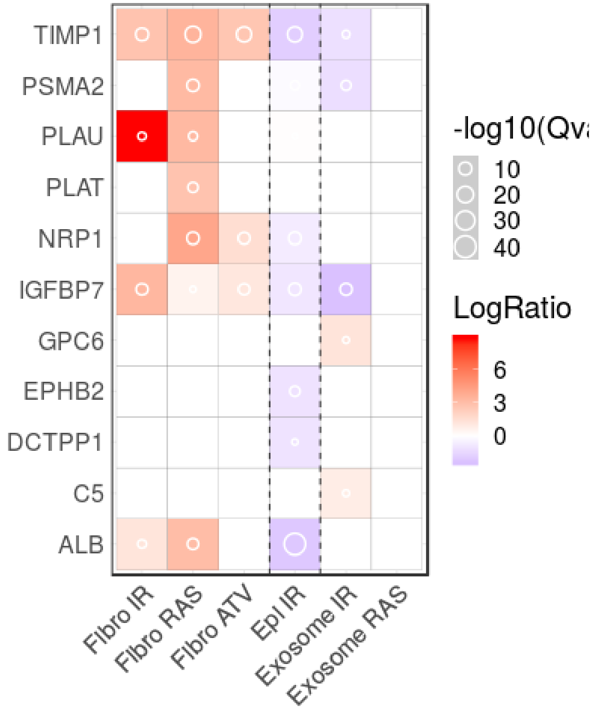

SASP proteins in the 50-protein signature of extreme old-age and their secretion level

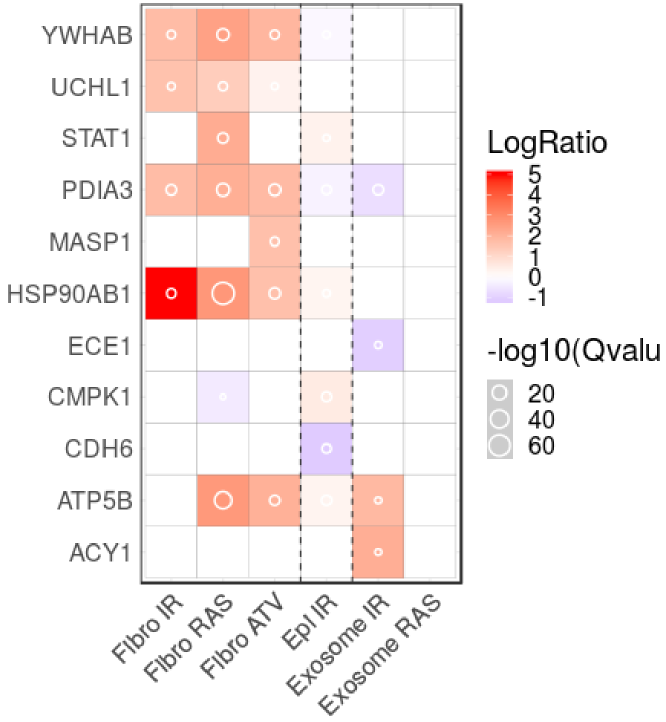

SASP proteins in the 37-protein signature of longer survival in centenarians

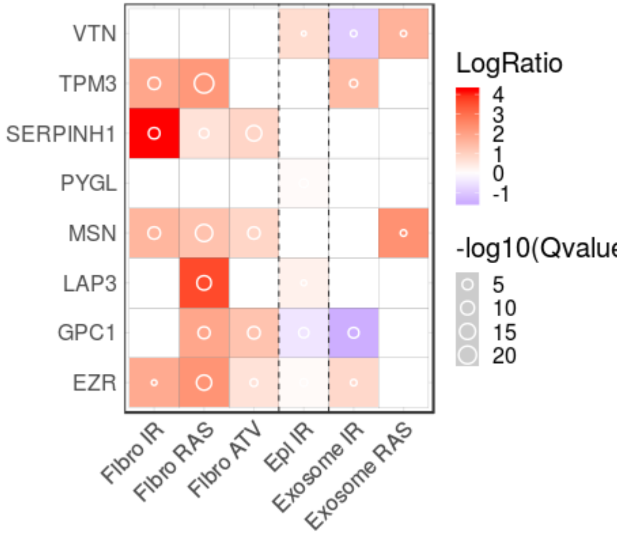

SASP proteins in the 140-protein signature of longer survival in older adults

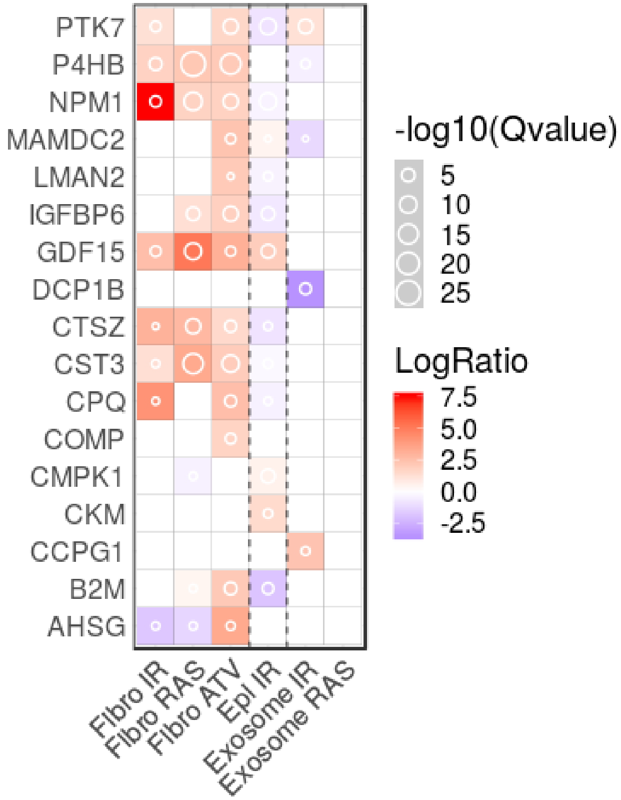

Supplement Figure 6

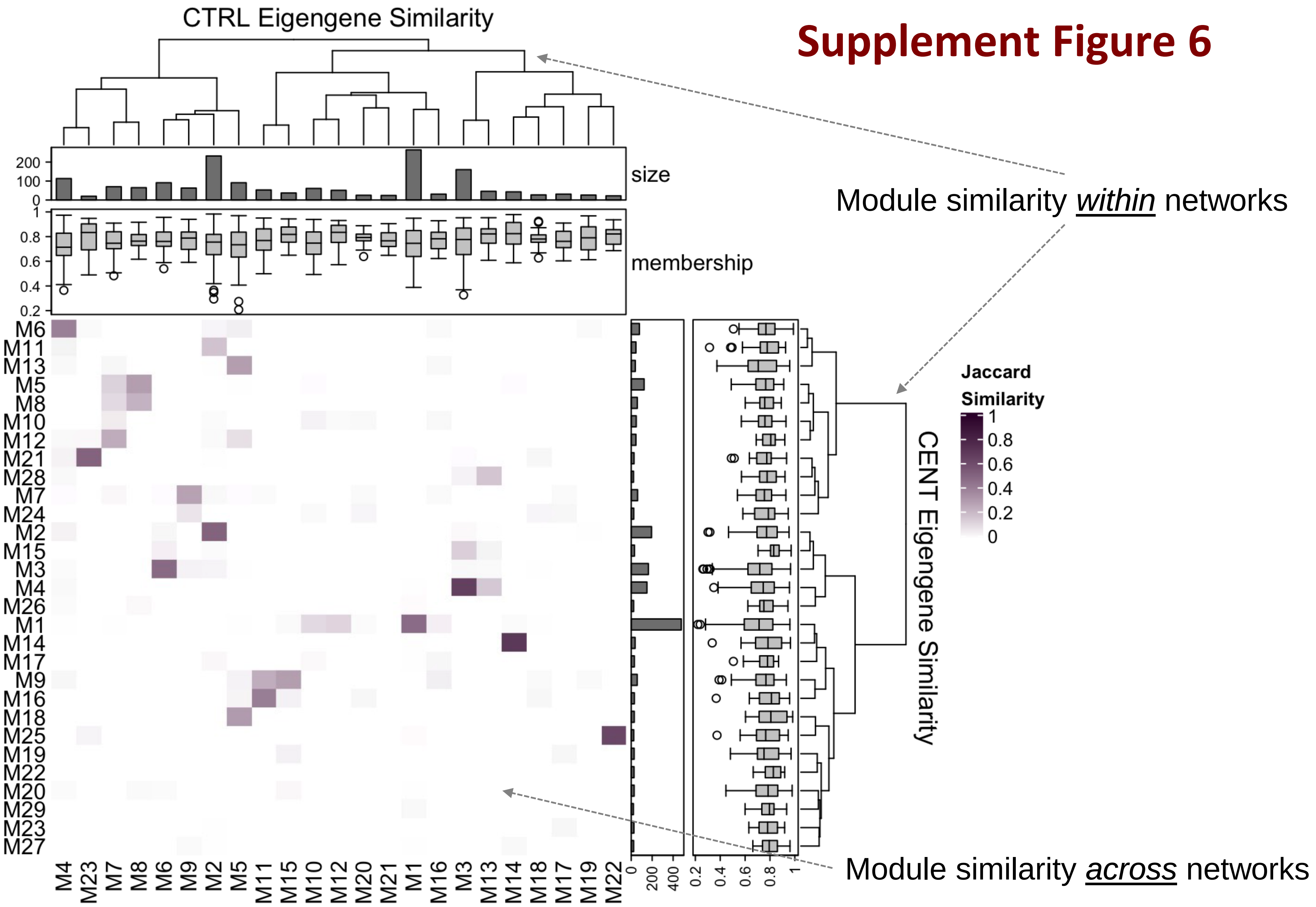

Supplement Figure 7

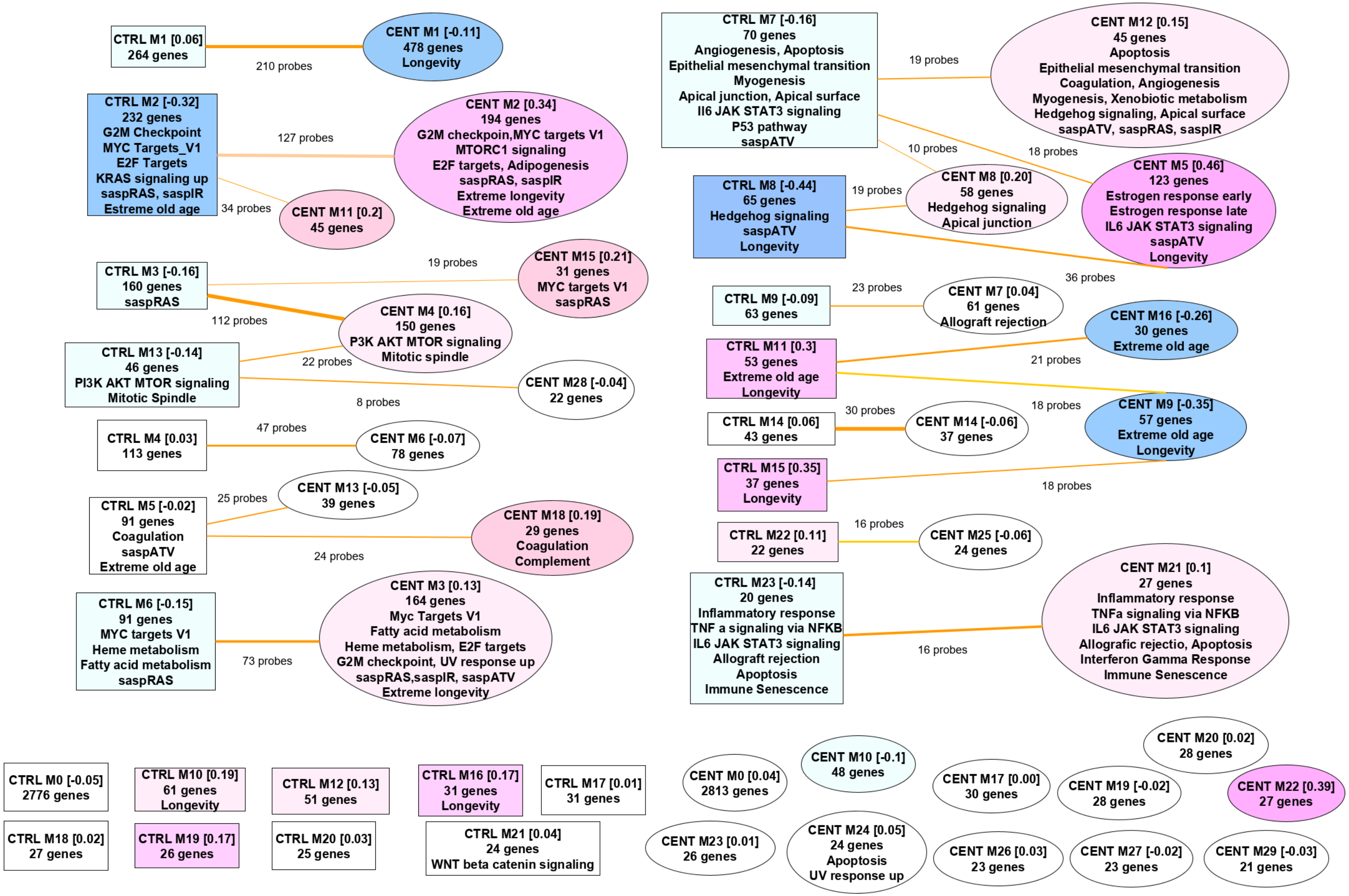

Supplement Figure 8

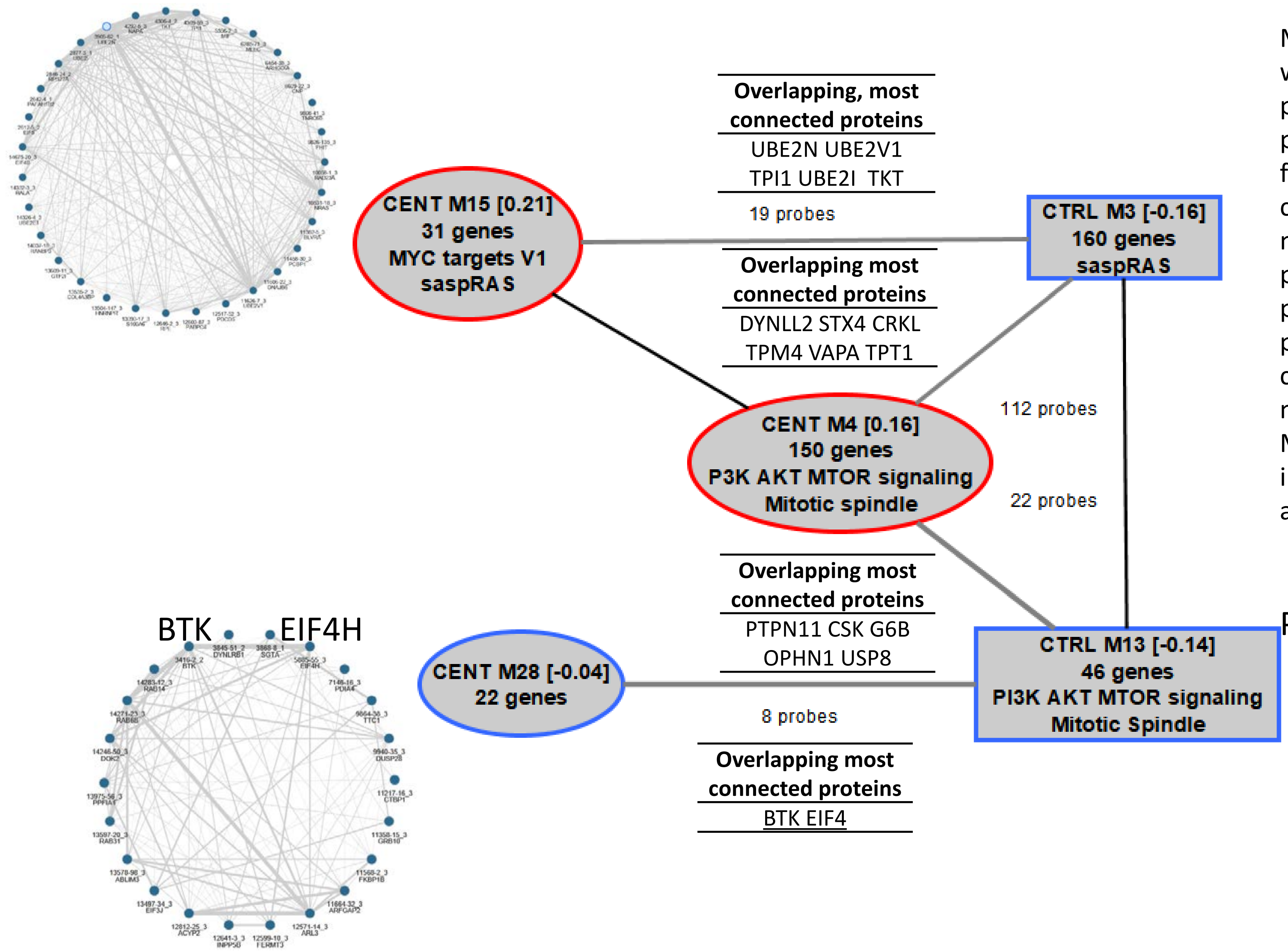

Module M3 in offspring and controls overlapped with 2 modules in centenarians: M4 that included proteins enriched for the P13/AKT/mTOR signaling pathway, and M15 that included proteins enriched for Myc targets and SASP markers. Module M4 in centenarians also overlapped with the much smaller module M13 in offspring and controls that clustered proteins enriched for the P13 AKT mTOR signaling pathway. CRKL activates the RAS kinase signaling pathway and two SASP proteins: TPT1, part of the centenarian signature, and TPM4. The overlapping most connected proteins in centenarians' modules M15 and offspring/controls module M3 were involved in protein degradation (UBE2N, UBE2V1, and UBE2I) and senescence (TKT).

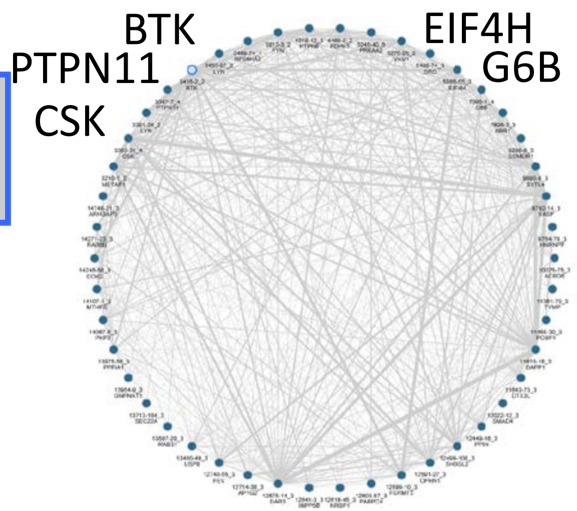

Supplement Figure 9

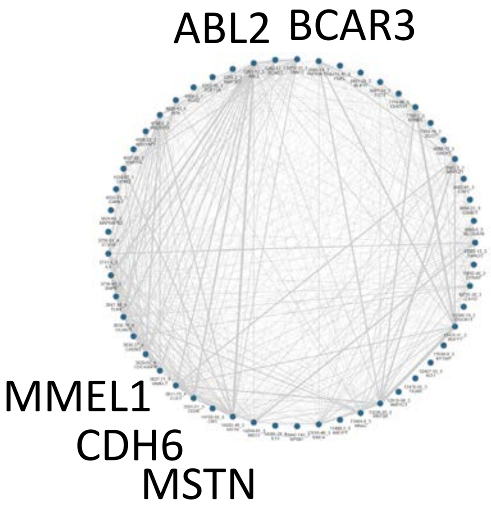

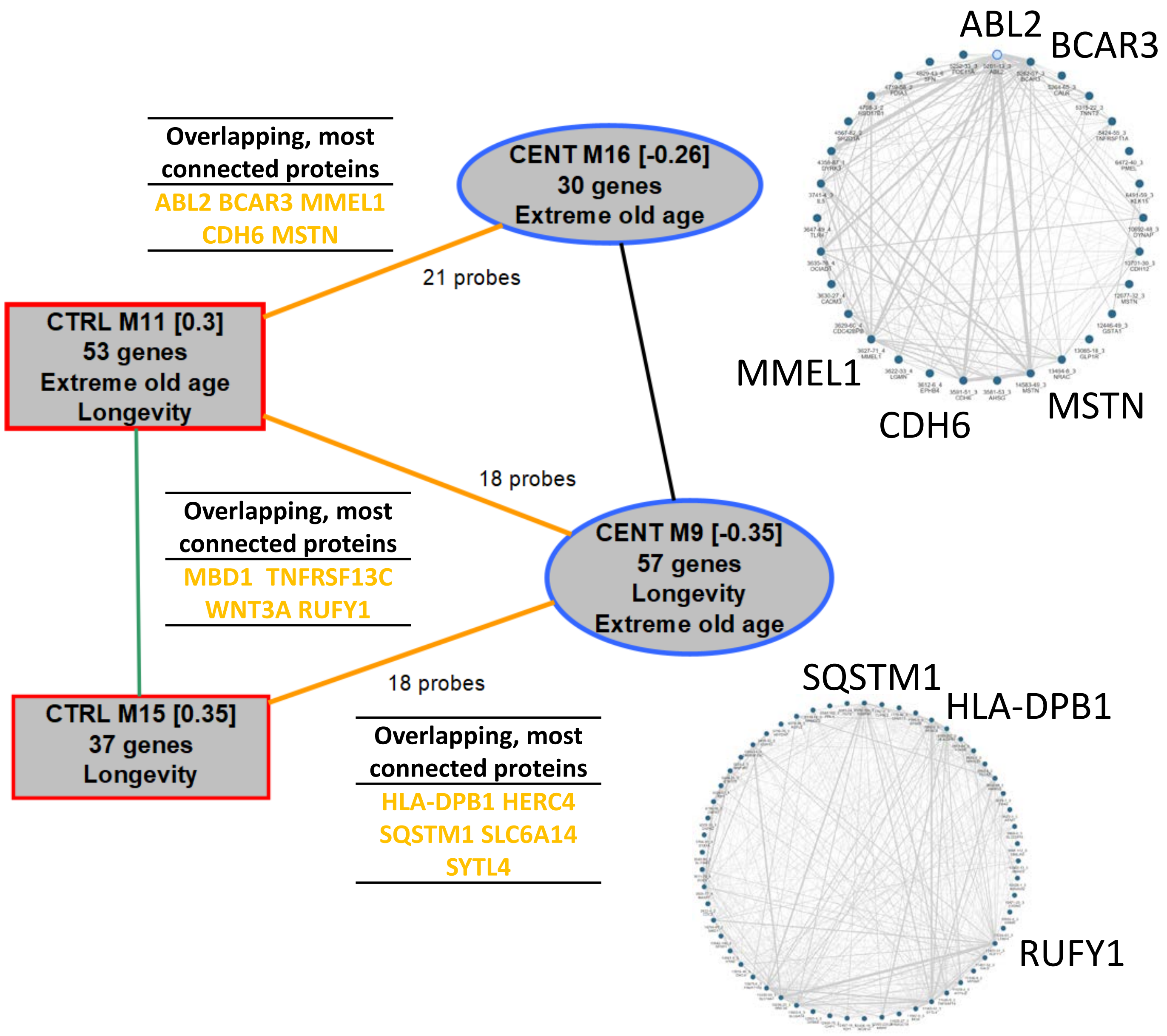

Module M16 in centenarians included 29 down-regulated proteins that were enriched only for the extreme old age signature. 70% of these proteins were also in the offspring and controls Module 11 that clustered 53 upregulated proteins enriched for both the extreme old age and longevity signatures. Module M15 in offspring and controls included 37 upregulated proteins, enriched for the survival signature, and 48% of these were also in modules M9 in centenarians that clustered 57 down-regulated proteins enriched for both signatures.
