# Supplementary material for: Protein signatures of centenarians and their offspring suggest centenarians age slower than other humans
Source: Aging Cell. 2021 Jan 29;20(2):e13290. doi: 10.1111/acel.13290 (PMC7884029; doi:10.1111/acel.13290)
Supplement: Supplementary file 1 — Appendix S1 [file ACEL-20-e13290-s001.zip › sm_0019-FileS1.docx]

**Methods**

**Data:** We used serum and plasma samples from 4 independent studies of aging and longevity.

*New England Centenarian Study (NECS):* The NECS is a study of centenarians, their long-lived siblings, offspring, and controls selected as either spouses of centenarian offspring or individuals whose parents died before reaching age 73-74 (1). The study began by recruiting centenarians in the Boston metropolitan area in 1994 and expanded in the late 1990s to include all of North America. The age of participants is carefully validated (2) and participants are followed-up annually to assess their health through self-reported medications, hospitalizations, and physical and cognitive functions that are assessed through questionnaires that are administered over the phone. We selected 226 participants (79 centenarians, 82 offspring, and 65 controls) who survived at least one year after the blood draw and were free of major aging-related diseases within 1 year from blood draw, and not treated with medications for serious medical conditions including cancer or diabetes, and serum samples were used for the protein scan. All subjects provided informed consent approved by the Boston University Medical Campus IRB.

*Baltimore Longitudinal Study of Aging (BLSA) and Genetic and Epigenetic Signatures of Translational Aging Laboratory Testing (GESTALT):* Plasma samples from 240 healthy men and women were selected from the BLSA and the GESTALT. The BLSA is a population‐based study of older adults that began in 1958 to discover factors that affect physiological and functional trajectories of aging. Participants are followed at intervals from 1 to 4 years, depending on their age (3). The GESTALT study began in 2015 to discover novel molecular biomarkers of aging (<https://www.nia.nih.gov/alzheimers/clinical-trials/genetic-and-epigenetic-signatures-translational-aging-laboratory-testing>). Both BLSA and GESTALT recruited healthy participants, ages 20 years or older, from the DC/Baltimore metropolitan area. Samples used in this study were selected at enrollment for GESTALT participants, and at times when all healthy criteria were still met for BLSA participants. Patients were selected to have 48 samples in five 15 year age categories (20-35, 35-50, 50-65, 65-80, 80+). Their characteristics are described in (4).

*InChianti*: This is a community-based cohort study with 1453 residents of Greve in Chianti (a rural area: 11,709 residents with 19.3% of the population older than 65 years of age) and Bagno a Ripoli (Antella village near Florence; 4,704 inhabitants, with 20.3% older than 65 years of age), selected from the population registry among people aged 21 and 102. Details of the study have been previously reported (5). Overnight fasted plasma samples were used proteomic assessment. For 997 subjects, proteomic profiles for 1322 SOMAmers were assessed using the 1.3k SOMAscan Assay at the Trans-NIH Center for Human Immunology, Autoimmunity, and Inflammation (CHI), National Institute of Allergy and Infectious Diseases, National Institutes of Health (Bethesda, MD, USA) using the same methods as previously published (6, 7). Mortality data were evaluated for up to 20 years following the baseline visit (1998-2000), assessed using the mortality general registry maintained by the Tuscany Region and through death certificates submitted to the Registry office of the municipality of residence immediately after death (8). The study protocol was approved by the Italian National Institute of Research and Care of Aging Institutional Review and Medstar Research Institute (Baltimore, MD) and approved by the Internal Review Board of the National Institute for Environmental Health Sciences (NIEHS). All participants provided written informed consent.

**Somascan technology.** The Novartis custom-designed aptamer profiling platform (Novartis Somascan©) was used at SomaLogic Inc. (Boulder, US) to measure protein levels, as previously described (9-11). The 226 serum samples from the NECS biorepository were assayed with 4,783 SOMAmers. The samples were randomized into analytical batches of 84 samples or less and the plates were assayed as a set, to avoid biases from technical procedures and sample processing. The Somascan results passed a quality control assessment for median intra- and inter-assay variability, CV ≤ 15%, similar to variability previously reported in the Somascan assays (12). Proteomic profiles of 240 plasma samples from BLSA and GESTALT were assayed using the 1.3K Somascan Assay at the Trans‐NIH Center for Human Immunology and Autoimmunity, and Inflammation (CHI), National Institute of Allergy and Infectious Disease, National Institutes of Health (Bethesda, MD, USA). Full details are in (4). Proteomic concentration of plasma sample is consistent with the relative abundance of SOMAmer reagents. The data readout from the Somascan-based proteomics is relative fluorescence units (RFUs) and is directly proportional to the reported relative abundances of the targets of the SOMAmer Reagents.

**Mass-spectrometry**.

*Reagents and Serum Samples.* Serum samples were obtained from the New England Centenarian Study and selected from the original pilot sample to cover the age range 50 to 100 uniformly. Trypsin/Lys-C Mix, Mass Spec Grade was obtained from Promega (Madison, WI, USA). UPLC grade acetonitrile, trifluoroacetic acid, and water, as well as the TMT10plex Isobaric Label Reagent Set plus TMT11-131C Label Reagent, Pierce Quantitative Colorimetric Peptide Assay kit, Pierce C18 spin columns, and Pierce Top 12 Abundant Protein Depletion Spin Columns, were purchased from Thermo Fisher Scientific (Waltham, MA, USA). Ammonium hydroxide solution (28-30% w/v), sodium deoxycholate, 2-chloroacetamide, ethyl acetate, and Amicon Ultra-0.5 mL Centrifugal Filters (3K NMWL) were purchased from Millipore Sigma (Burlington, MA, USA). A 1M solution of Tris, pH 8.5 was purchased from K D Medical (Columbia, MD, USA). LoBind microcentrifuge tubes were purchased from Eppendorf (Hamburg, Germany).

*Sample Preparation.* Serum samples from individuals homozygous for ApoE3 (E3/E3, n=10) were subject to depletion of abundant proteins followed by trypsin/LysC digestion. A pooled serum sample, composed of equal volumes of the 10 donor serum samples, was used as a reference for all analyses. First, a 10 µL aliquot of each serum sample was depleted using Pierce™ Top 12 Abundant Protein Depletion Spin Columns (Thermo Fisher Scientific, Waltham, MA, USA), according to the manufacturer’s instructions. Following depletion, samples were concentrated using Amicon Ultra-0.5 mL Centrifugal Filters (3K NMWL, Millipore Sigma, Burlington, MA, USA) to a volume ≤ 50 µL, by spinning at 14,000 RCF for 30 min. Next, 2X concentrated reduction and alkyalation buffer was added to each sample to achieve a final concentration of 1% w/v sodium deoxycholate (SDC), 10 mM TCEP, 40 mM 2-chloroacetamide, and 100 mM Tris pH 8.5. Samples were heated for 10 min at 95 °C to denature proteins, then allowed to cool to room temperature. Trypsin/LysC was added at a 1:100 enzyme-to-protein ratio, and digestion was performed at 37 °C for 4 hr. Peptides were acidified to a final concentration of 0.5% trifluoroacetic acid (TFA). Samples were briefly spun (14000 RCF, 5 min) to pellet precipitated sodium deoxycholate, and the supernatant was transferred to new tubes. Peptides were desalted based on procedures adapted from Geyer PE *et al*., 2016 (13). Briefly, peptides were added to Pierce C18 spin columns, and ethyl acetate with 1% TFA was used to remove sodium deoxycholate. Peptides were eluted in 80% acetonitrile, 19% ddH2O, 1% ammonia and dried under vacuum. Peptide concentrations were determined using the Pierce Quantitative Colorimetric Peptide Assay kit, followed by measurement of the absorbance at 480 nm on a Synergy HT microplate reader (BioTek, Winooski, VT, USA). TMT labeling was performed according to the manufacturer’s protocol, and quenched with 5% hydroxylamine. Following the quenching step, samples were pooled and dried under vacuum, followed by resuspension in 500 µL 1% acetonitrile/99% water + 0.1% formic acid, desalted using a SepPak C18 1cc cartridge (elution: 80% acetonitrile/20% water + 0.1% formic acid) , and dried under vacuum. High pH reversed phase separation was performed using a gradient generated with ddH_2_O, 1% acetonitrile, 0.1% NH_4_OH (buffer A) and ddH_2_O, 80% acetonitrile, 0.1% NH_4_OH (buffer B). Peptides were separated and eluted from 5% B to 62.5% B in 30 min, and fractions were collected at 30 s. intervals. Fractions were subsequently dried under vacuum, then suspended in 1% acetonitrile/99% water + 0.1% formic acid, for nUPLC-MS/MS.

*nUPLC-MS/MS Analysis of Peptides from Serum Digests.* Peptides were analyzed on an Orbitrap Fusion Lumos Tribrid mass spectrometer (Thermo Scientific) equipped with an ACQUITY UPLC M-Class system (Waters) and a TriVersa NanoMate (Advion). For chromatographic separation, a nanoEase Symmetry C18 UPLC Trap Column (100 Å, 5 μm, 180 μm × 20 mm, Waters) was used for trapping, and a nanoEase MZ HSS C18 T3 UPLC Column (100 Å, 1.8 μm, 75 μm × 100 mm, Waters) was used for separation. The peptide trapping step was performed at 4 μL/min for 4 min with 1% acetonitrile and 0.1% formic acid (Solvent A). Following the trapping step, peptides were separated on the analytical column according to the following conditions: 0–1 min: 2% B, 1–3 min: 2–5% B, 3–43 min: 5–30% B (Solvent B: 99% acetonitrile and 0.1% formic acid). All analyses were performed in positive mode, with the RF lens set to 30%. Ions were analyzed in the Orbitrap for MS1 scans with 120,000 resolution @ *m/z* 400, scan range *m/z* 400-1200, 1 μscan/MS, AGC target 2.0 x 10^5^, and a maximum injection time of 100 ms. Data-dependent acquisition was performed. Ions were filtered as follows: monoisotopic peak determination (MIPS) was set to the peptide mode, charge states 2-7 were considered, and a dynamic exclusion time of 60 sec. was enabled after one fragmentation event, and a minimum intensity threshold of 3.0 x 10^4^. A maximum of eight data-dependent scans were performed for each cycle. For HCD MS2, ions were isolated in the quadrupole prior to fragmentation using an isolation window of 0.7 *m/z.* Following fragmentation (33% collision energy), ions were analyzed in the Orbitrap with the following settings: 50,000 resolution @ *m/z* 400, scan range *m/z* 100–2000, 1 μscan/MS, AGC target 5 x 10^4^, and a maximum injection time of 86 ms. Profile spectra were recorded. A second fragmentation event (MS3) was performed using synchronous precursor selection with the following additional settings: 10 precursors were selected from MS2, MS isolation window of 2.5 *m/z*, MS2 isolation window of 3.0 *m/z*, 55% collision energy, 50,000 resolution @ *m/z* 400, AGC target 5.0 x 10^4^, 1 μscan/MS, detection in the Orbitrap, and a maximum injection time of 80 ms.

*Peptide Assignment.* Data analysis was performed using PEAKS Studio v8.5 (Bioinformatics Solutions Inc., Waterloo, ON, Canada). The following search parameters were used for MS2 spectra: 10.0 ppm parent mass error tolerance, fragment mass error tolerance 0.02 Da, Trypsin-LysC (cleavage at K, R), up to 3 missed cleavages, and TMT11plex (229.16) and carbamidomethylation (C) (fixed modifications). A database consisting of reviewed protein sequences from Uniprot *Homo sapiens* UP000005640 (downloaded Sept. 29, 2019), was used for all searches. Filtration criteria for protein matches included 1% false discovery rate, and ≥ 1 unique peptide.

**Statistical Analyses.**

*Quality Control*: We used principal component analysis of data and side-by-side boxplots of sample expression to search for residual batch effects and outlier samples. This analysis (**Supplement Figure 1**) identified two samples of poor quality that we removed and 224 samples were included in the remaining analyses. All analytes data were log-transformed and, for each analyte, values that differed by more than three standard deviations from the mean were removed. We used regression of the first principal component to test the hypothesis that the age of serum samples (time since blood collection) has an effect on the analyte level, adjusting for sex and experimental groups. We did not see a significant effect of serum sample age on the variability of data (p=0.21).

*Centenarian signature.* We used linear regression analysis of the log-transformed RFU of each analyte versus the groups centenarians, offspring and controls. The variable group was coded as a factor with three levels and centenarians were chosen as the referent group. The analysis was adjusted by sex and year of sample collection, fitting the regression equation:

$$E\left( y_{j} \right)=\beta_{0}+\beta_{c} X_{c,j}+\beta_{o} X_{o,j}+\beta_{sex} X_{sex,j}+\beta_{l} X_{l,j}$$

Where $y_{j}$ is the log-transform RFU in sample $j$, $X_{c.j}$ is an indicator variable taking on value 1 when sample $j$ is a control and 0 otherwise, $X_{o.j}$ is an indicator variable taking on value 1 when sample $j$ is a centenarian offspring and 0 otherwise, $X_{sex,j}$ denotes sex and $X_{l,j}$ is the length of sample $j$ storage. Note that $exp(\beta_{c})$ and $exp(\beta_{0})$ represent the fold change of expression comparing controls to centenarians, and offspring to centenarians respectively.. We selected differentially expressed proteins that differ between offspring and centenarians, or between control and centenarians, using the F-test on 2 and 219 degrees of freedom, with false discovery rate at 1% and Benjamini Hochberg correction (14). We used in house scripts for analysis using linear regression and also the limma package in R for a sensitivity analysis. This analysis selected 1428 analytes (1313 unique gene symbols). A similar analysis was used to compare protein expression between controls and centenarians’ offspring, by fitting the modified regression equation:

$$E\left( y_{j} \right)=\beta_{0}+\beta_{c} X_{c,j}+\beta_{sex} X_{sex,j}+\beta_{l} X_{l,j}$$

Where $X_{c.j}$ is an indicator variable taking on value 1 when sample $j$ is a control and 0 otherwise. We did not use a linear regression of protein versus age at blood draw because often age-related changes are not linear. See for example reference (15).

*Signature of Survival.* To distinguish between aging and longevity, we analyzed the protein expression data using an ANOVA based on two factors: age at blood draw, and additional years of survival after blood draw. We did not correlate the protein data to survival using survival models because of the small number of deaths observed in the younger age group, but we conducted two different analyses separating the centenarians and offspring generations because of the wide age gap between the two groups and difference in survival time. In centenarians, we removed 3 individuals with age < 100, and defined strata of age at blood draw (100-104; 105-109; 110+) to avoid a linear effect of age on survival, and of length of survival after the blood draw (< 2 years, 2 years and longer). We choose two years, since this is approximately the average number of years of life expected in centenarians based on the 1900 cohort table from the US social security administration (16). We then used linear regression of the log-transformed RFU of each aptamer versus the follow-up group, adjusting for sex, and age strata at blood draw that is a strong predictor or mortality, fitting the regression equation:

$$E\left( y_{j} \right)=\beta_{0}+\beta_{g} X_{g,j}+\beta_{sex} X_{sex,j}+\beta_{age,i} X_{age i,j}$$

Where $y_{j}$ is the log-transform RFU in sample $j$, $X_{g.j}$ is an indicator variable taking on value 1 when sample $j$ has shorter survival and 0 otherwise, $X_{sex,j}$ denotes sex and $X_{age i,j}$ is the age stratum of sample $j$ at blood draw. Note that $exp(\beta_{g})$ represents the fold change of expression comparing centenarians who survived less than 2 years to those who survived longer than 2 years. We selected aptamers that are significantly associated with length of survival using the F-test on 1 and 69 degrees of freedom (p < 0.005). In the younger generation comprised of offspring and controls, we generated two strata of age at blood draw (70 years or younger and 71 and older) to avoid linear modeling of age on mortality, and survival up to 10 years, or greater than 10 years. Note that 10 years is approximately the average number of years of life expected in individuals aged 70 years and born in the 1930 cohort, which is comparable to offspring and controls used in this analysis. We used linear regression of log-transformed RFU versus survival group, adjusted by sex, age strata at blood draw, and an indicator variable denoting whether individuals were children of centenarians or controls, by fitting the regression equation:

$$E\left( y_{j} \right)=\beta_{0}+\beta_{g} X_{g,j}+\beta_{sex} X_{sex,j}+\beta_{age,i} X_{age i,j}+\beta_{o} X_{o,j}$$

Where $y_{j}$ is the log-transform RFU in sample $j$, $X_{g.j}$ is an indicator variable taking on value 1 when sample $j$ has shorter survival and 0 otherwise, $X_{sex,j}$ denotes sex, $X_{age i,j}$ is the age stratum of sample $j$ at blood draw, and $X_{o,j}$ is an indicator variable taking value 1 if sample $j$ is a centenarian offspring. Note that in this analysis $exp(\beta_{g})$ represents the fold change of expression comparing individuals who survived less than 10 years to those who survived longer than 10 years. We selected analytes that are significantly associated with length of survival using the F-test on 1 and 64 degrees of freedom (p < 0.005).

*Gene sets pathways analyses.* To analyze the association of known gene signatures with aging and longevity, we performed geneset (or pathway) projection analysis of all proteins in the Somascan array, whereby the protein-by-sample data was mapped to a geneset-by-sample data, with each entry denoting the level of activity of a given geneset (pathway) in a given sample. We selected 2 geneset compendia available through [MSigDB](http://software.broadinstitute.org/gsea/msigdb/index.jsp) (http://software.broadinstitute.org/gsea/msigdb/index.jsp): the “Canonical Pathways” compendium [C2.CP](mailto:http://software.broadinstitute.org/gsea/msigdb/genesets.jsp?collection=CP), and the “[Hallmarks](http://software.broadinstitute.org/gsea/msigdb/genesets.jsp?collection=H)” compendium, which comprises sets of genes defining specific well-defined biological states or processes and displaying coherent expression. Since there are only 4116 unique gene names represented in the Somascan assay, we first reduced the genesets to the overlapping genes, and eliminated the genesets with less than 5 members. This step produced 1061 and 50 genesets from the C2.CP and Hallmarks compendia, respectively. On these lists, we used the gene set variation analysis method (GSVA) implemented in the R package gsva (17). In particular, GSVA uses a modified Kolmogorov-Smirnov test to generate an enrichment score for each subject and each gene set. The enrichment scores are in the range [-1.0;+1.0], with a positive (negative) score indicating coordinated up- (down-) regulation in the sample. We visualized the enrichment scores using heatmaps. We then conducted differential analysis comparing centenarians vs. controls and offspring aggregated in one single group using linear regression adjusted by sex, and year of sample collection, and selected pathways with FDR < 5%. We also analyzed differential pathway scores comparing centenarians with shorter survival to centenarians with longer survival, as well as offspring/controls with shorter survival to offspring/controls with longer survival (p-value < 10%).

*Replication and Meta-Analysis with the BLSA/GESTALT and inChianti studies.* We identified 1291 analytes common to both the platforms used to profile the NECS serum samples and the BLSA/GESTALT plasma samples. In the *BLSA/GESTALT*, protein expression was correlated to chronological age using a linear regression model of log-transformed RFU versus age adjusting for sex, study (BLSA or GESTALT), race (White, Black, other), and technical covariates (plateID). To have comparable estimates in the NECS proteomic data, we estimated the age effect in year as $b_{\mathrm{cent}}/35$ where $b_{\mathrm{cent}}$ is the centenarian effect compared to the younger groups from the ANOVA model, and 35 is the difference of mean age between centenarians and offspring/controls. We similarly estimated the standard error as ${SE(b}_{\mathrm{cent}})/35$. (**Supplement Figure 3**). We used fixed effect meta-analysis to aggregate the results from the two studies, using the function metagen from the meta package in R. Significant results were selected based on 1% FDR that was computed using the Benjamini Hochberg correction (14). Since wedid not have mortality data in the BLSA/GESTALT study, survival associated proteins were replicated using Cox-proportional hazard regression, adjusted by sex and age at blood draw in inChianti.

*Annotation of protein lists*. We used Hyper to annotate significant proteins by their function, using the list of proteins in the array as background (18), and the program String v.11 for visualization of known protein-protein relations (19).

*Comparison to transcriptional profiles in whole blood***.** UniProt IDs from the protein study were converted to Entrez gene IDs using DAVID (<https://david.ncifcrf.gov>). The set of common genes between this study (n=4074) and Peters et al (n=11730) was used as a background list (n=2590). Significantly up-regulated and down-regulated genes from each study were separately tested for overlaps (overlap of n=43 in up-regulated genes; n=25 in down-regulated genes). No functional enrichment was detected in either the overlapping up regulated or down regulated genes when tested with hypeR (github.com/montilab/hyper, (18) ) against the MSigDB Hallmark collection (Supplementary Tables 1,2). Alternate analysis with g:ProfileR (Reimand, J. et al., Nucleic Acids Res. ,2016) produced similar results (Supplementary Tables 3,4).

*Correlation of protein abundance with age using MS data*. We removed peptide intensity data linked to any of the 12 depleted proteins (ALBUMIN, APOA1, APOA2, CRP, A1AG1, A1AG2, A1AT, A2MG, HPT, FIB, IGH, TRFE), and rescaled the remaining data to the median intensity per sample. We analyzed the cubic-root transformation of the normalized data using a Bayesian hierarchical model adjusted for batch, in which we modeled the age effect on total protein abundance using random effects for different peptides that were normally distributed with a common mean (fixed effect) and standard deviation. The fixed age effect for each protein was estimated using the median of a Markov Chain Monte Carlo sample of size 2,500 that was generated with the R-package Rjags after a burn-in of 500 iterations. Concordance of effects from the two analyses was analyzed in Rstudio.

**Correlation network analysis.**

*Co-expression Modules***.** Network co-expression modules were identified for 4783 aptamers using the WGCNA package (version 1.68.0) (20) for both centenarians (n = 77) and controls (n = 147) separately, whereby the control group included both control patients as well as centenarian offspring. An appropriate soft-thresholding power for the correlation values was selected from the value range 1-10 (for which the scale free topology fit indices were calculated) for each group using the *pickSoftThreshold* function. Hierarchical clustering was performed on the topological overlap dissimilarity matrix using the “average” agglomeration method, and modules were selected using the “tree” method, a cut height of 0.995, and a minimum cluster size of 20. The minimum cluster size selected was the default recommended by the tool, which is based on extensive application of the methods in multiple studies. Additionally, in our attempt to prevent an excessive number of modules, we opted for a high cut height of the dendrogram, choosing 0.995, which is between the default of 0.99 and the max. Similar modules, identified by the correlation of their “eigengenes”*,* were merged using the *mergeCloseModules* function and a cut height of 0.15. We performed evaluation of alternative cluster sizes ranging from 5 to 50, to evaluate the robustness of the attained results. While results marginally changed for different cluster sizes, the main results were consistent throughout, with the smaller cluster sizes yielding additional modules of difficult interpretation, and larger sample sizes missing some important module distinctions, as assessed by enrichment-based annotation.

*Module Eigengene.* The eigengene for each module was calculated by using the first principle component of the expression data of a given modules’ members across samples.

*Module Membership.* For each module, a membership value was assigned to each member. Module membership was calculated by taking the absolute value of the correlation of a members’ expression with the module eigengene.

*Connections between Modules.* Connections between modules were measured by the correlation between modules’ eigengenes. Modules with correlation > 0.85 (**Supplement Figure 5)** were connected in the network in **Figure 4**.

*Matching Modules.* Modules for centenarians and controls were matched to identify similar co-expression modules arising within both groups. The Jaccard index (21) was calculated for each centenarian-control pair of modules. Module pairs with a similarity >= 0.1 were considered matched.

*Module Quality.* We determined module quality by generating Z-scores for various network statistics introduced by WGCNA through permutation-based testing, including variance explained by module eigengene, average correlation with module eigengene, and average correlation, among others. Additionally, we considered *Z-Summary*, which is a composite of multiple metrics, providing an overall quality measure (22). Based on the summary and individual metrics, we found both centenarian and control modules to be well-defined with Z-scores above 10, indicating high robustness (22). These additional analyses are included in the supplement document Module-Statistics-and-Quality.html.

*Module Enrichment.* Pathway enrichment of modules was performed with the hypeR package (version 1.1.9) (18). Aptamers were converted to gene symbols and enrichment was tested using the “hypergeometric” method. The background population size was set to the number of unique gene symbols (4,100), and the tested genesets were reduced by taking their overlap with the background population of unique genes.

Essential analysis scripts will be shared via [https://GitHub.com/montilab](https://github.com/montilab)

**Supplement Figures.**

**Supplement Figure 1. Quality Control Steps.** Principal component analysis left), side-by-side boxplots of log2 proteins RFU by samples middle) and boxplot of the median protein expression for each sample, with the two outlying samples in red. The first two principal components explained 94% of the total variability. The median expression of the two samples in red differed by more than the 1^st^ quartile -1.5* the interquartile range, and the 3^rd^ quartile + 1.5* the interquartile range were detected and the two samples were removed from the subsequent analyses. The heatmap in the right panel shows the complete set of 224 samples and 4785 analytes that passed the quality control steps. The heatmap displays the normalized intensities by analytes (rows, sorted by hierarchical clustering) and samples (columns, sorted by hierarchical clustering). The “barcoding” at the bottom shows the 3 comparisons groups sorted by hierarchical clustering: centenarians cluster in a group that is different from the other 3 groups, suggesting that a large number of proteins may be linked to aging.

**Supplement Figure 2. Flow chart of the analysis conducted in the manuscript.**

**Supplement Figure 3. Concordance of age effects of the aptameters in common between the Somascan arrays used in the NECS and BLSA/GELSTALT studies.** A) The scatter plots show the agreement between the estimates of the age effects for all 1291 aptamers that were measured in both NECS and BLSA/GELSTALT studies. The set of 1291 aptamers includes fewer proteins that decrease with older age in the BLSA study compared to the platform used in the NECS. The age effects in NECS are on average bigger, as shown by the plots above and below the diagonal. Scatter plots in panels b) and c) show the agrrement for subsets of the aptamers that were significant in NECS (panel b) and BLSA/GELSTALT (panel c).

**Supplement Figure 4.** Summary of the comparison between the Interval, BLSA/GESTALT and NECS Studies**.**  A) Agreement of the statistical association with age of 2,317 aptamers common to the NECS and the Interval studies. The agreement of the significant associations with age of 453 aptamers (<1%FDR) was statistically significant (p from Fisher exact test 1.471e-10). B) Cross-classification of the significant and not-significant associations between the NECS and the Interval studies. The groups “Age up” and “Age down” include aptamers that correlate positively and negatively with age. The groups “Cent up”, “Cent flat”, and “Cent down” denote proteins that are higher/not different/lower in centenarians compared to offspring/controls. The two columns in yellow provide a breakdown of the 453 associations in the table in the A) panel. The column in pink provides a breakdown of the 296 associations in the table in the A) panel. C) Venn diagram of the overlap between the results of the three studies.

**Supplement Figure 5. Overlap with SASPs.** List of SASP proteins in the signatures of immune senescence, extreme old age, extreme longevity and longevity. The plots were generated using the SASPatlas portal (<http://www.saspatlas.com/>)**.**

**Supplement Figure 6. Module similarity within networks.**  A summary of module overlaps and network statistics for both CENT and CTRL groups. The heatmap represents module similarity across groups measured by the Jaccard Index, while module similarity within groups is represented by hierarchical clustering of module eigengenes. Additionally, module statistics such as size and boxplots of module membership values are included.

**Supplement Figure 7. Connections between modules discovered in centenarians and offspring/controls data.** Squares denote protein modules in centenarians’ offspring and controls (CTRL) and ellipses denote protein modules in centenarians (CENT). Pink nodes denote upregulated modules and blue nodes denote down-regulated modules. Orange edges represent protein overlap between centenarian modules and centenarians’ offspring and controls modules. The numbers within square brackets represent the average standardized expression comparing CTRL to CENT in the CTRL modules and the other way around in CENT modules. For example, CENT M1 [-0.11] means that the average standardized expression of the proteins in the module comparing centenarians to offpring/control is -0.11. Each module was annotated with enrichment for the 50 hallmark pathways, the 32-protein signature of immune senescence, the 50-protein signature of extreme old age, the 37-protein signature of extreme longevity, the 140-protein signature of longevity, and the 3 SASP signatures of senescence.

**Supplement Figure 8. Details of the overlap between modules M15, M4, M28 in centenarians and modules M3 and M13 in offspring and controls.** Squares denote protein modules in centenarians’ offspring and controls (CTRL) and ellipses denote protein modules in centenarians (CENT). A red border denotes upregulated modules and a blue border denotes down-regulated modules. Grey edges represent correlations between centenarians’ offspring and controls modules, and black edges represent correlation between centenarians’ modules. The numbers within square brackets represent the average standardized expression comparing CTRL to CENT in the CTRL modules and the other way around in CENT modules. Edges are annotated by the overlapping, most connected proteins as shown in the circular graphs that represent the within module connections among proteins.

**Supplement Figure 9. Details of the overlap between modules M9, M16 in centenarians and modules M11 and M15 in offspring and controls.** Squares denote protein modules in centenarians’ offspring and controls (CTRL) and ellipses denote protein modules in centenarians (CENT). A red border denotes upregulated modules and a blue border denotes down-regulated modules. Grey edges represent correlations between centenarians’ offspring and controls modules, green edges represent correlation between centenarians’ modules, and orange edges represent the overlap between modules. The most connected proteins annotate the orange edges as shown in the circular graphs that represent the within-module connections among proteins.

**Supplement Tables.**

**Supplement Table 1: a) Centenarian Signature.** Results of differential expression analysis using ANOVA, adjusted for sex and serum storage time. FC cont to cent: Fold change comparing protein abundance in controls versus centenarians (Note that FC cont to cent > 1 indicates a protein that decreases in centenarians, while FC cont to cent < 1 indicates a protein that increases in centenarians). FC off to cent: Fold change comparing protein abundance in centenarians’ offspring versus centenarians (FC off to cent > 1 indicates a protein that decreases in centenarians, while FC off to cent < 1 indicates a protein that increases in centenarians). Pvalue F-test: p-value from ANOVA F-test to compare the three groups (centenarians, centenarians’ offspring and controls), adjusted for sex and serum storage time. Adjusted p-values were computed using the Benjamini, Hochberg correction. **b) Serum biomarkers selected by the TAME consortium and their difference in centenarians and younger controls**. FC cont to cent: Fold change comparing protein abundance in controls versus centenarians. The adjusted p-value were reported from Supplement Table 1a). c) **list of genes overexpressed and underexpressed in blood that overlap with the proteins in the aging signature**. The gene list was derived from (23). d) **Comparison of correlation with age in 443 proteins measured with mass-spectrometry in the serum of 10 samples included in the Somalogic experiment**. No peptide = number of peptides associated with the protein; MEAN, SD and pval = mean and standard error of the age effect on the protein abundance in the MS analysis; pval= p-value to test the hypothesis of no correlation between the protein and age. Columns O-T are results of the aptamers in Supplement Table 1a. “in aging” indicates whether the aptameter was included in the aging signature. Flag indicates whether the protein was significant in the MS-based analysis. Concordance flags the concordant effects of the two analysis. e) **List of 59 aptamers that are significantly different between centenarians offspring and controls and are also in the aging signature**. Columns A—G are from Supplement Table 1a. FC.contr.Offs is the fold change of protein abundance comparing controls to centenarians’ offspring. Pval is the raw p-value from the regression analysis, and adjusted p-values were computed using the Benjamini, Hochberg correction.

**Supplement Table 2**. Functional annotation from DAVID of the centenarian signature [1428 aptamers corresponding to 1312 unique proteins].

**Supplemental Table 3**. Differential analysis of pathway projection scores (a: hallmark gene sets; b: C2Cp). Estimate is the differential score comparing offspring/controls to centenarians.

**Supplement Table 4a**. Replication of the results from Santos-Lozano et al (24).

**Supplement Table 4b**. Replication of the results from Menni et al (25).

**Supplement Table 4c: Signature of Immune Senescence**. List of proteins significantly associated with aging in the BLSA/GESTALT study (FDR< 1%) and not significant in the centenarian study (p > 0.20). Beta, se, p: regression coefficient of log-transformed protein data versus chronological age, standard error and p-value in BLSA/GESTALT analysis. Beta.age, beta.age.se and pval.Ftest: regression coefficient of log-transformed protein data versus chronological age, standard error and p-value in NECS analysis.

**Supplement Table 4d: Signature of Extreme Old Age**. List of proteins significantly associated with aging in the NECS study (FDR< 1%) and not significant in the BLSA/GESTALT study (p > 0.20). Beta, se, p: regression coefficient of log-transformed protein data versus chronological age, standard error and p-value in BLSA/GESTALT analysis. Beta.age, beta.age.se and pval.Ftest: regression coefficient of log-transformed protein data versus chronological age, standard error and p-value in NECS analysis.

**Supplement Table 4e**. Meta-analysis of results common to NECS and BLSA/GESTALT studies. Beta, se, p: regression coefficient of log-transformed protein data versus chronological age, standard error and p-value in BLSA/GESTALT analysis. Beta.age, beta.age.se and pval.Ftest: regression coefficient of log-transformed protein data versus chronological age, standard error and p-value in NECS analysis. beta.meta, se.meta, p-meta: regression coefficient of log-transformed protein data versus chronological age, standard error and p-value from meta-analysis. sign.necs =1 if significant in discovery set at 1%FDR; sign.blsa=1 if significant in BLSA/GESTALT analysis at 1%FDR; sign.meta=1 if significant in meta-analysis at 1%FDR.

**Supplement Table 5a**. List of 296 proteins with significant correlation with age in the Interval study, but no change in centenarians’ serum. FC cont to cent: Fold change comparing protein abundance in controls versus centenarians. FC off to cent: Fold change comparing protein abundance in centenarians’ offspring versus centenarians. Pvalue F-test: p-value from ANOVA F-test to compare the three groups (centenarians, centenarians’ offspring and controls), adjusted for sex and serum storage time. Beta.age, SE.age and log.10.p. are regression coefficient, standard error and -log10-pvalue from the Interval study.

**Supplement Table 5b**. List of 347 aptamer with concordant and significant effects in the NECS and the Interval study. Columns headers as in Supplement Tables 4d and 5a.

**Supplement Table 5c**. List of 502 aptamers in the aging signature that replicate in either the BLSA/GESTALT or the Interval studies.

**Supplement Table 6a: Signature of Survival in Centenarians**. List of 37 aptamers associated with differential survival in NECS centenarians. log(FC comparing long to short survival): Fold change in log-scale of protein abundance comparing individuals with longer survival (> 2 years) versus shorter survival. Columns from aging signature analysis are from Supplement Table 1.

**Supplement Table 6b**. Annotation of the Extreme Longevity Signature using the 50 Hallmark pathways. Estimate is the differential expression comparing short versus longer survival.

**Supplement Table 7: Signature of Survival in Centenarians’ Offspring and Controls**. List of 140 aptamers associated with differential survival in NECS centenarians’ offspring and controls. Log(FC long vs short survival): Fold change in log-scale of protein abundance comparing individuals with survival > 10 years versus less than or equal to 10 years. Columns from aging signature analysis are from Supplement Table 1.

**Supplement Table 8.** SASP markers in the various signatures.

**Supplement Table 9.** Module compositions in centenarians (CENT) and offspring/controls (CTRL) discovered with the network co-expression analysis.

**Supplement Table 10**. Summary of annotation of all aptamers.

**Additional References**

1. Sebastiani P, Perls TT. The genetics of extreme longevity: lessons from the New England Centenarian Study. Front Genet. 2012;3:277. Epub 2012/12/12. doi: 10.3389/fgene.2012.00277. PubMed PMID: 23226160; PMCID: 3510428.

2. Young RD, Desjardins B, McLaughlin K, Poulain M, Perls TT. Typologies of extreme longevity myths. Curr Gerontol Geriatr Res. 2010;2010:423087. Epub 2010/01/01. doi: 10.1155/2010/423087. PubMed PMID: 21461047; PMCID: 3062986.

3. Stone JL, Norris AH. Activities and attitudes of participants in the Baltimore longitudinal study. J Gerontol. 1966;21(4):575-80. PubMed PMID: 5918312.

4. Tanaka T, Biancotto A, Moaddel R, Moore AZ, Gonzalez-Freire M, Aon MA, Candia J, Zhang P, Cheung F, Fantoni G, consortium CHI, Semba RD, Ferrucci L. Plasma proteomic signature of age in healthy humans. Aging Cell. 2018:e12799. doi: 10.1111/acel.12799. PubMed PMID: 29992704.

5. Ferrucci L, Bandinelli S, Benvenuti E, Di Iorio A, Macchi C, Harris TB, Guralnik JM. Subsystems contributing to the decline in ability to walk: bridging the gap between epidemiology and geriatric practice in the InCHIANTI study. J Am Geriatr Soc. 2000;48(12):1618-25. doi: 10.1111/j.1532-5415.2000.tb03873.x. PubMed PMID: 11129752.

6. Tanaka T, Biancotto A, Moaddel R, Moore AZ, Gonzalez-Freire M, Aon MA, Candia J, Zhang P, Cheung F, Fantoni G, consortium CHI, Semba RD, Ferrucci L. Plasma proteomic signature of age in healthy humans. Aging Cell. 2018;17(5):e12799. doi: 10.1111/acel.12799. PubMed PMID: 29992704; PMCID: PMC6156492.

7. Osawa Y, Semba RD, Fantoni G, Candia J, Biancotto A, Tanaka T, Bandinelli S, Ferrucci L. Plasma proteomic signature of the risk of developing mobility disability: A 9-year follow-up. Aging Cell. 2020:e13132. Epub 2020/03/12. doi: 10.1111/acel.13132. PubMed PMID: 32157804.

8. Zuliani G, Volpato S, Dugo M, Vigna GB, Morieri ML, Maggio M, Cherubini A, Bandinelli S, Guralnik JM, Ferrucci L. Combining LDL-C and HDL-C to predict survival in late life: The InChianti study. PLoS One. 2017;12(9):e0185307. Epub 2017/09/29. doi: 10.1371/journal.pone.0185307. PubMed PMID: 28957382; PMCID: PMC5619755.

9. Hathout Y, Brody E, Clemens PR, Cripe L, DeLisle RK, Furlong P, Gordish-Dressman H, Hache L, Henricson E, Hoffman EP, Kobayashi YM, Lorts A, Mah JK, McDonald C, Mehler B, Nelson S, Nikrad M, Singer B, Steele F, Sterling D, Sweeney HL, Williams S, Gold L. Large-scale serum protein biomarker discovery in Duchenne muscular dystrophy. Proc Natl Acad Sci U S A. 2015;112(23):7153-8. doi: 10.1073/pnas.1507719112. PubMed PMID: 26039989; PMCID: PMC4466703.

10. Davies DR, Gelinas AD, Zhang C, Rohloff JC, Carter JD, O'Connell D, Waugh SM, Wolk SK, Mayfield WS, Burgin AB, Edwards TE, Stewart LJ, Gold L, Janjic N, Jarvis TC. Unique motifs and hydrophobic interactions shape the binding of modified DNA ligands to protein targets. Proc Natl Acad Sci U S A. 2012;109(49):19971-6. doi: 10.1073/pnas.1213933109. PubMed PMID: 23139410; PMCID: PMC3523867.

11. Emilsson V, Ilkov M, Lamb JR, Finkel N, Gudmundsson EF, Pitts R, Hoover H, Gudmundsdottir V, Horman SR, Aspelund T, Shu L, Trifonov V, Sigurdsson S, Manolescu A, Zhu J, Olafsson O, Jakobsdottir J, Lesley SA, To J, Zhang J, Harris TB, Launer LJ, Zhang B, Eiriksdottir G, Yang X, Orth AP, Jennings LL, Gudnason V. Co-regulatory networks of human serum proteins link genetics to disease. Science. 2018. doi: 10.1126/science.aaq1327. PubMed PMID: 30072576.

12. Candia J, Cheung F, Kotliarov Y, Fantoni G, Sellers B, Griesman T, Huang J, Stuccio S, Zingone A, Ryan BM, Tsang JS, Biancotto A. Assessment of Variability in the SOMAscan Assay. Sci Rep. 2017;7(1):14248. doi: 10.1038/s41598-017-14755-5. PubMed PMID: 29079756; PMCID: PMC5660188.

13. Geyer PE, Kulak NA, Pichler G, Holdt LM, Teupser D, Mann M. Plasma Proteome Profiling to Assess Human Health and Disease. Cell Syst. 2016;2(3):185-95. doi: 10.1016/j.cels.2016.02.015. PubMed PMID: 27135364.

14. Benjamini Y, Hochberg Y. Controlling the false discovery rate - a practical and powerful approach to multiple testing. J Roy Stat Soc B Met. 1995;57(1):289-300. PubMed PMID: ISI:A1995QE45300017.

15. Sebastiani P, Thyagarajan B, Sun F, Honig LS, Schupf N, Cosentino S, Feitosa MF, Wojczynski M, Newman AB, Montano M, Perls TT. Age and sex distributions of age-related biomarker values in healthy older adults from the long life family study. J Am Geriat Soc. 2016;64(11):e189-e94. doi: 10.1111/jgs.14522.

16. Bell FC, Miller ML Life Tables for the United States Social Security Area 1900-2100. In: , Office of the Chief Actuary Social Securty Administration, editor.: SSA Pub. No. 11-11536. Washington, DC; 2005.

17. Hanzelmann S, Castelo R, Guinney J. GSVA: gene set variation analysis for microarray and RNA-seq data. BMC Bioinformatics. 2013;14:7. doi: 10.1186/1471-2105-14-7. PubMed PMID: 23323831; PMCID: PMC3618321.

18. Federico A, Monti S. hypeR: an R package for geneset enrichment workflows. Bioinformatics. 2020;36(4):1307-8. doi: 10.1093/bioinformatics/btz700. PubMed PMID: 31498385.

19. Szklarczyk D, Gable AL, Lyon D, Junge A, Wyder S, Huerta-Cepas J, Simonovic M, Doncheva NT, Morris JH, Bork P, Jensen LJ, Mering Christian v. STRING v11: protein–protein association networks with increased coverage, supporting functional discovery in genome-wide experimental datasets. Nucleic Acids Research. 2018;47(D1):D607-D13. doi: 10.1093/nar/gky1131.

20. Langfelder P, Horvath S. WGCNA: an R package for weighted correlation network analysis. BMC Bioinformatics. 2008;9(1):559. doi: 10.1186/1471-2105-9-559.

21. Sebastiani P, Perls TT. Detection of Significant Groups in Hierarchical Clustering by Resampling. Frontiers in Genetics. 2016;7(144). doi: 10.3389/fgene.2016.00144.

22. Langfelder P, Luo R, Oldham MC, Horvath S. Is my network module preserved and reproducible? PLoS Comput Biol. 2011;7(1):e1001057. doi: 10.1371/journal.pcbi.1001057. PubMed PMID: 21283776; PMCID: PMC3024255.

23. Peters MJ, Joehanes R, Pilling LC, Schurmann C, Conneely KN, Powell J, Reinmaa E, Sutphin GL, Zhernakova A, Schramm K, Wilson YA, Kobes S, Tukiainen T, Consortium NU, Ramos YF, Goring HH, Fornage M, Liu Y, Gharib SA, Stranger BE, De Jager PL, Aviv A, Levy D, Murabito JM, Munson PJ, Huan T, Hofman A, Uitterlinden AG, Rivadeneira F, van Rooij J, Stolk L, Broer L, Verbiest MM, Jhamai M, Arp P, Metspalu A, Tserel L, Milani L, Samani NJ, Peterson P, Kasela S, Codd V, Peters A, Ward-Caviness CK, Herder C, Waldenberger M, Roden M, Singmann P, Zeilinger S, Illig T, Homuth G, Grabe HJ, Volzke H, Steil L, Kocher T, Murray A, Melzer D, Yaghootkar H, Bandinelli S, Moses EK, Kent JW, Curran JE, Johnson MP, Williams-Blangero S, Westra HJ, McRae AF, Smith JA, Kardia SL, Hovatta I, Perola M, Ripatti S, Salomaa V, Henders AK, Martin NG, Smith AK, Mehta D, Binder EB, Nylocks KM, Kennedy EM, Klengel T, Ding J, Suchy-Dicey AM, Enquobahrie DA, Brody J, Rotter JI, Chen YD, Houwing-Duistermaat J, Kloppenburg M, Slagboom PE, Helmer Q, den Hollander W, Bean S, Raj T, Bakhshi N, Wang QP, Oyston LJ, Psaty BM, Tracy RP, Montgomery GW, Turner ST, Blangero J, Meulenbelt I, Ressler KJ, Yang J, Franke L, Kettunen J, Visscher PM, Neely GG, Korstanje R, Hanson RL, Prokisch H, Ferrucci L, Esko T, Teumer A, van Meurs JB, Johnson AD. The transcriptional landscape of age in human peripheral blood. Nat Commun. 2015;6:8570. doi: 10.1038/ncomms9570. PubMed PMID: 26490707; PMCID: PMC4639797.

24. Santos-Lozano A, Valenzuela PL, Llavero F, Lista S, Carrera-Bastos P, Hampel H, Pareja-Galeano H, Gálvez BG, López JA, Vázquez J, Emanuele E, Zugaza JL, Lucia A. Successful aging: insights from proteome analyses of healthy centenarians. Aging. 2020;12(4):3502-15. doi: 10.18632/aging.102826.

25. Menni C, Kiddle SJ, Mangino M, Vinuela A, Psatha M, Steves C, Sattlecker M, Buil A, Newhouse S, Nelson S, Williams S, Voyle N, Soininen H, Kloszewska I, Mecocci P, Tsolaki M, Vellas B, Lovestone S, Spector TD, Dobson R, Valdes AM. Circulating Proteomic Signatures of Chronological Age. J Gerontol A Biol Sci Med Sci. 2015;70(7):809-16. doi: 10.1093/gerona/glu121. PubMed PMID: 25123647; PMCID: PMC4469006.
